# Differential response of soil microbial and animal communities along the chronosequence of *Cunninghamia lanceolata* at different soil depth levels in subtropical forest ecosystem

**DOI:** 10.1016/j.jare.2021.08.005

**Published:** 2021-08-11

**Authors:** Waqar Islam, Hafiz Sohaib Ahmad Saqib, Muhammad Adnan, Zhenyu Wang, Muhammad Tayyab, Zhiqun Huang, Han Y.H. Chen

**Affiliations:** a Institute of Geography, Fujian Normal University, Fuzhou 350007, China; b Key Laboratory for Humid Subtropical Eco-Geographical Processes of the Ministry of Education, Fujian Normal University, Fuzhou 350002, China; c Post-doctoral Research Station of Geography, Fujian Normal University, Fuzhou, 350007, China; d STU-UMT Joint Shellfish Research Laboratory, Shantou University, Shantou 515063, China; e College of Life Sciences and Oceanography, Shenzhen University, Shenzhen 518060, China; f College of Agriculture, Fujian Agriculture and Forestry University, Fuzhou 350002, China; g Faculty of Natural Resources Management, Lakehead University, Ontario, Canada

**Keywords:** Microbial diversity, Soil animals, Soil microbiome, Soil nutrients, Tropical forest, Vertical profiles, TC, Total carbon, TN, Total nitrogen, TP, Total phosphorus, AP, Available phosphorus, TK, Total potassium, AK, Available potassium, BD, Bulk density, NO_3_, Nitrate, WC, Water-holding capacity, OTUs, Operational taxonomic units, ANOVA, Analysis of variance, LSD, Least Significant Difference, ANOSIM, Analysis of similarity, VIFs, Variation inflation factors, NMDS, Non-metric multidimensional scaling, RDA, Redundancy analysis

## Abstract

**Introduction:**

Soil biota plays a crucial role in the terrestrial ecosystem. There is growing momentum to understand the community structure and diversity of total belowground soil biota across large ecological scales. Soil biota follow divergent trends with respect to soil physiochemical properties in different ecosystems; however, little is known about their response to stand development across multiple soil depths in Chinese fir plantations, which is the most important tree species across all over China, popular for its timber production.

**Objectives:**

Here, we investigated the community assembly of soil bacteria, fungi, archaea, protists and animals across three different vertical soil profiles (0–10, 10–20, 20–40 cm) using a chronosequence of Chinese fir representing five different stand ages (5, 8, 21, 27, 40 years) in South China.

**Methods:**

High throughput illumine Hiseq2500 sequencing.

**Results:**

Our results showed that soil biotic communities exhibited a decreasing trend in alpha diversity of bacteria, fungi, protists and animals with increasing soil depth; however, archaea showed an opposite trend. Most abundant soil bacterial, fungal, archaeal, protist and animal classes were Acidobacteriia, Agaricomycetes, Bathyarchaeia, Chlorophyceae and Clitellata, respectively. Correlation of vertical distribution of biotic communities and variations in soil physiochemical properties explained that total nitrogen (TN), available phosphorus (AP) and pH were the most influencing factors for changes in soil biotic communities. Although the stand age was a contributing factor for fungal and animal beta diversity, however, as compared to soil depth, it was not dominatingly influencing the structure of other biotic communities.

**Conclusions:**

Collectively, these results reveal a new perspective on the vertical variation and distinct response patterns of soil biotic communities at a fine scale across different stand ages of Chinese fir plantations.

## Highlights

• We investigated the assembly of soil bacteria, fungi, archaea, protists & animals across different soil depths in five stand ages.
• Soil biotic communities exhibited a decreasing trend in alpha diversity with increasing soil depth.
• Acidobacteriia, Agaricomycetes, Bathyarchaeia, Chlorophyceae and Clitellata, were most abundant classes.
• Total nitrogen, available phosphorus and pH were the most influencing factors for changes in soil biotic communities.
• As compared to soil depth, stand age was not dominatingly influencing the structure of other biotic communities.

## Graphical abstract

**Figure:**
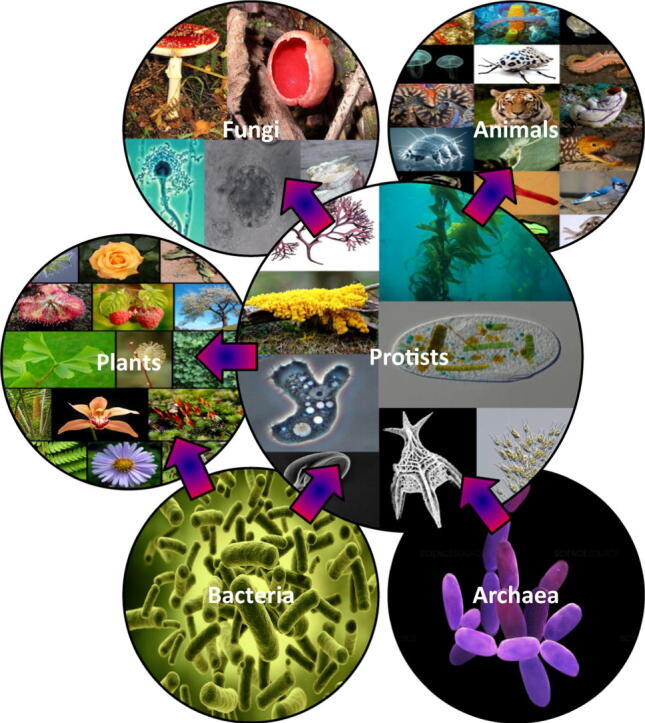

## Introduction

Chinese fir (*Cunninghamia lanceolata* (Lamb.) Hook.] is an ancient fast-growing, evergreen coniferous tree, popular for its good timber quality and high yield, particularly in subtropical China. The tree is cultivated for commercial purposes, as its timber is utilized to construct houses and furniture manufacturing. In recent years, the area under Chinese fir plantation has increased rapidly in China [1]. Apparently, this has happened due to the increasing demand for wood products because of rapid population increase and economic development in the country. Usually, the harvesting of mature Chinese fir timber stands is done at the age of 25–30 years; however, recently the harvesting time is shortened (20–25 years) to fulfill the high demands [2].

The repetitive plantation of fast-growing species at the same site can decline soil fertility [3]. Significant decline in timber yield and soil fertility due to repetitive monoculture Chinese fir plantations have already been well documented [4], [5]. Soil nutrient deficiency [6], auto-toxicity of root exudates [7], reduction in biochemical activity [2] and alterations in the population of soil biotic communities are categorized as the main reasons for the decline in timber yield [8].

Soil biotic communities constitute prokaryotes and eukaryotes that may be further divided into soil microbial (Bacteria, Fungi, Archaea, Protists), animal and plant communities [9]. A fundamental role of soil biotic communities include nutrient and hydrological cycling, litter decomposition and supporting terrestrial primary production [10], [11]. Literature highlights that stand age in monoculture forests [12], [13] and soil depth/vertical profiles greatly impact soil biotic communities [14]. Soil organisms are strongly influenced by changes in the soil ecosystem and alteration in available nutrients [15]. Moreover, soil biotic communities’ response is accepted as an ecological indicator for soil quality [16]. Previous reports indicate that successive plantations of Chinese fir on the same site led to a significant decrease in nitrogen fixation (%), ammonification, fiber decomposition, respiration (%), soil pH, organic matter, humus carbon, C/N-ratio, total and available nitrogen, potassium and phosphorus [2], [5], which likely altered the composition, structure, and diversity of soil biotic communities [8].

Nevertheless, soil arbuscular mycorrhizal fungi composition is recently attributed to the chronosequence of Chinese fir using high-throughput sequencing on an Illumina Miseq platform [17]. Similarly, another recent study evaluated the soil microbial (Fungi & Bacteria) community diversity along with three different rotations of 26 years old Chinese fir plantations [8]. However, despite strong interactions among soil organisms [18], the response of all the soil biotic communities (Bacteria, Fungi, Archaea, Protists and Animals) to different stand ages and soil vertical profiles across the chronosequence of Chinese fir has not been previously studied.

High-throughput sequencing technology has shown great advantages in understanding the soil biotic diversity and community composition because of its unprecedented sequencing depth [19]. Therefore, in this study, we employed the high throughput (Illumina Hiseq2500) sequencing to assess the composition of soil microbial (Bacteria, Fungi, Archaea, Protists) and animal communities across a chronosequence of Chinese fir plantations at five different stand ages (5, 8, 20, 27, 40 years) with three different vertical soil depth levels (0–10, 10–20, 20–40 cm). We assumed that Chinese fir plantations with different stand ages might result in shifts among soil physiochemical properties, microbial and animal communities at various vertical soil depth profiles. Specifically, we aimed to understand that **(a)** How soil microbial (Bacteria, Fungi, Archaea, Protists) and animal diversity and community composition changes between different Chinese fir plantation stages/ages at various vertical soil profiles? **(b)** How the changes in soil microbial and animal communities’ structure are correlated with environmental factors across various vertical soil profiles and different stand ages of Chinese fir?

## Material and methods

### Experimental sites, sampling and soil analysis

Our experiment site was located at Baisha forest farm (25°15′ N, 116°61′ E, altitude 1181 ft) near Longyan city (Southwestern China, Fujian Province), which is a well-managed forest site between Baopinling and Nanzhao mountains (Wuyi mountain range). The climate is monsoon-influenced humid subtropical (Köppen climate classification), having mild short winter, long hot and humid summer. Average annual precipitation is 1738 mm while average daily precipitation is 162 mm (frost-free period-280 days) with a mean annual temperature of 20.4° C (highest in July i.e., 29. 2° C; lowest in January i.e., 6.8° C). The mean evapotranspiration is 1598 mm, and the annual mean relative humidity is 75%. According to IUSS Working Group-WRB-2014, the soils in the Longyan area belongs to Oxisol (thickness > 62 cm), developed from granite [20]. Historically, our experimental forest area was impacted by typhoons and consecutive intensive harvesting. For Chinese fir monoculture plantations, the site was prepared by removing logging residues and additional vegetation, followed by establishing the contoured strips and digging of plant holes. The plantation of seedlings (3600 stems/ha) was done leading towards the application of synthetic fertilizers (N: 54, P_2_O_5_: 54, K_2_O: 54 kg/ha per seedling). Weeds and other competing vegetation removal processes were performed manually on a regular basis for the first three years of seedling establishment and growth. Furthermore, the standard thing process was followed at the ages of 8, 18, and 25 years.

To understand the comparative composition and diversity of total belowground soil microbiota with relation to soil physiochemical properties in Chinese fir plantations, we employed a chronosequence approach to collect the soil samples among each stand. Chronosequence approach has been previously criticized by assuming that the sample stands may follow a similar development history along a temporal sequence [21]. However, careful site selection and replications enable us to investigate the ecological changes / processes on yearly, decadal, continental and time scales basis [22]. Soil samples among five age chronosequences were done in June 2019. We selected five different ages (5, 8, 21, 27, and 40 years) to represent different classes i.e., early stand initiation, canopy closure, stem exclusion, canopy transition, and gap dynamics, respectively [23]. We selected visually homogenous stands (>1 km distance among stands) on the basis of their structure, composition and spatial distribution across large areas for minimizing the impact of spatial autocorrelation. For each sampled stand, plots of 20 m × 30 m were assigned randomly, which were at least 100 m away from the forest edge that abutted to agricultural lands, roads, and forests of different age classes. After removal of detritus and litter in each plot, we used 3.8 cm auger to collect soil samples (Bulk soil) from ten random locations to prepare a composite sample by mixing. A total of 45 composite samples from three soil depths (0–10, 10–20, 20–40 cm) were collected. After the removal of loose gravel, stones, and plant debris, the samples were stored in plastic bags with ambient field moisture content at 4° C, labelled and transported to the laboratory for downstream experimentation.

All the samples were sieved through ≤ 2 mm mesh and were divided into two portions, with one immediately stored –80 °C for molecular analysis, while the other was air-dried for measurement of soil physiochemical properties as quantified previously [24] including pH (measured in 0.01 CaCl_2_), total carbon (TC- g/kg), total nitrogen (TN- g/kg), C/N-ratio, total phosphorus (TP- mg/kg), available phosphorus (AP- mg/kg), total potassium (TK- g/kg), available potassium (AK- mg/kg), bulk density (BD- g/cm^3^), nitrate (NO_3_– mg/kg) and water-holding capacity (WC- g/kg).

### DNA extraction, primer selection, PCR and high-throughput sequencing

Extraction of genomic DNA was done through mechanical lysis followed by homogenization (triplicate) using 0.20 g soil from each sample by using the DNeasy PowerSoil Pro Kit (QIAGEN, USA). As our soil was acidic, the samples were pre-treated with 750 μL of 1 M CaCO_3_, to improve the PCR performances [25]. Extracted DNA was stored at − 20 °C till further preparation of amplicon library.

Amplicon libraries were generated using primers for rRNA marker genes, specifically for the V3 + V4 region of the 16S rDNA gene targeting bacteria and archaea (515F/806R)[26], ITS1 targeting fungi (ITS5/5.8S_Fungi)[27], and the V4 region of the 18S rDNA gene (TAReuk454FWD1/TAReukREV3)[28] targeting a wide range of eukaryotes (Protists, Animals and Plants), but not all eukaryotic organisms. The amplification of samples was performed in triplicate keeping no-template controls, followed by pooling of triplicate PCR amplicons and detection via electrophoresis in 2% (w/v) agarose gel [29]. High quality (bright bands) PCR products were mixed in equal ratio leading to purification using the GeneJET Gel Extraction Kit (Thermo Fisher Scientific). Final library preparation and Illumina Hiseq2500 (300 bp paired-end reads) sequencing was conducted at Bio-marker (Pvt) limited, Beijing. The obtained sequences were filtered by following a standard protocol [26]. USEARCH tool (UCHIME algorithm) was used to remove chimeric sequences, if any [30]. UPARSE pipeline was used for grouping the sequences and assigning the operational taxonomic units (OTUs) according to their taxonomy at a 3% dissimilarity level [30]. Non-classified OTUs at all taxonomic levels and the OTUs having less than two sequences were removed followed by classification of sequences according to Greengenes 13.8 [31], UNITE 7.2 [32], SILVA 128 [33] for 16S, ITS1, 18S, respectively. OTUs belonging to eukaryotes in the 16S OTU table, non-fungal OTUs in ITS OTU table, as well as fungal, non-soil animals and plant OTUs from the 18S OTU table were removed.

### Statistical analysis

The linear response of soil physiochemical properties at various soil depths and stand ages were assessed using linear regression model followed by the analysis of variance (ANOVA) and Fisher’s Least Significant Difference (LSD) *Post-hoc* tests [34]. The relative abundance of top 15 microbial and animal communities’ classes among all the samples was calculated using phyloseq [35] and microbiomeSeq [36] packages in R, followed by testing the linear response of each taxon at various age and depth. The Chao1 and Shannon-Wiener diversity indices were calculated in each sample, and their linear responses were measured at various soil depths and stand ages using linear regression models followed by ANOVA and the LSD *Post-hoc* tests. Beta-diversity was estimated via non-metric multidimensional scaling (NMDS) using the Bray–Curtis distance matrix between the samples. Moreover, perMANOVA (permutational multivariate analysis of variance) was used to model the influence of stand ages and soil depths on the Bray-Curtis dissimilarity matrix by applying the Adonis and analysis of similarity (ANOSIM) tests in vegan after 999 permutations [37]. Pearson’s correlation test was used to show the relationships of soil microbial and animal classes with soil physiochemical properties (AK, AP, BD, TC, C/N-Ratio, NO_3_, pH, TK, TN, TP and WC). Moreover, the associated *p*-values were also calculated and adjusted with the Benjamin and Hochberg procedure [38] to show the significance of each correlation. Heatmaps were drawn to show the correlations by following the R-codes provided by Torondel et al. [39].

A multivariate approach using redundancy analysis (RDA) was used to show each taxa's linear response to various environmental variables. Before performing RDA, community metrics were Hellinger transformed. This transformation enables us to use the ordination methods on datasets containing many zeros. We used variation inflation factors (VIFs) to test the goodness of fit to each RDA model. Since environmental variables with VIF > 10 had collinearity with other environmental variables [40], they do not significantly explain the model’s variance, and these were removed from our final model. Moreover, an ANOVA-like permutation test was used to test RDA models’ significance and environmental variables [41]. All calculations were conducted, and graphs were drawn in R (v-3.6.3), using “phyloseq” [35], ‘‘microbiomeSeq’’ [35], ‘‘ggplot2’’ [42], “vegan” [37], “dplyr”, “forcats”, “multcompView” and “extrafont” packages [43].

## Results

### Soil physiochemical properties

With the change in soil depth profiles and stand age, the soil physiochemical properties showed diverse trends (Supplementary Fig. S1). For example, TC and TN significantly decreased with the increase in soil depth (0–40 cm) (*p* < 0.001) (Supplementary Fig S1-A,B). A similar decreasing trend was observed for TP (*p* = 0.11), AP (*p* < 0.001), soil pH (*p* = 0.34) and NO_3_ (*p* = 0.05) (Supplementary Fig S1-D,E,F,I). Other soil physiochemical properties, such as C/N-Ratio, TK, AK, and BD exhibited variable patterns with increased soil depth. However, with respect to increasing age, TC, TN and AP showed an increase from 8 years to the age 27 significantly (*p* = 0.01), followed by a slightly decreasing trend up to age 40 years (Supplementary Fig S1-A). On the contrary, soil pH (*p* = 0.001) and NO_3_ (*p* = 0.001) exhibited a significant decrease up to 27 years followed by an increase in 40 years forest stand (Supplementary Fig S1-F,I). All other properties showed variable trends with respect to an increase in stand age.

### Distribution of soil microbial and animal communities

Among the entire samples, Illumina Hiseq2500 sequencing resulted in a total of 1,889,175 (Bacteria-16 s:V3 + V4), 3,557,061 (Fungi-ITS1), 1,737,854 (Archaea-16 s:V3 + V4), 196,257 (Protists-18 s:V4) and 909,546 (Animals-18 s:V4) clean paired reads, grouped into 1120 bacterial, 599 fungal, 99 archaea, 317 protist and 284 animal OTUs at 97% sequence similarity cutoff rate.

**Fig. 1:**
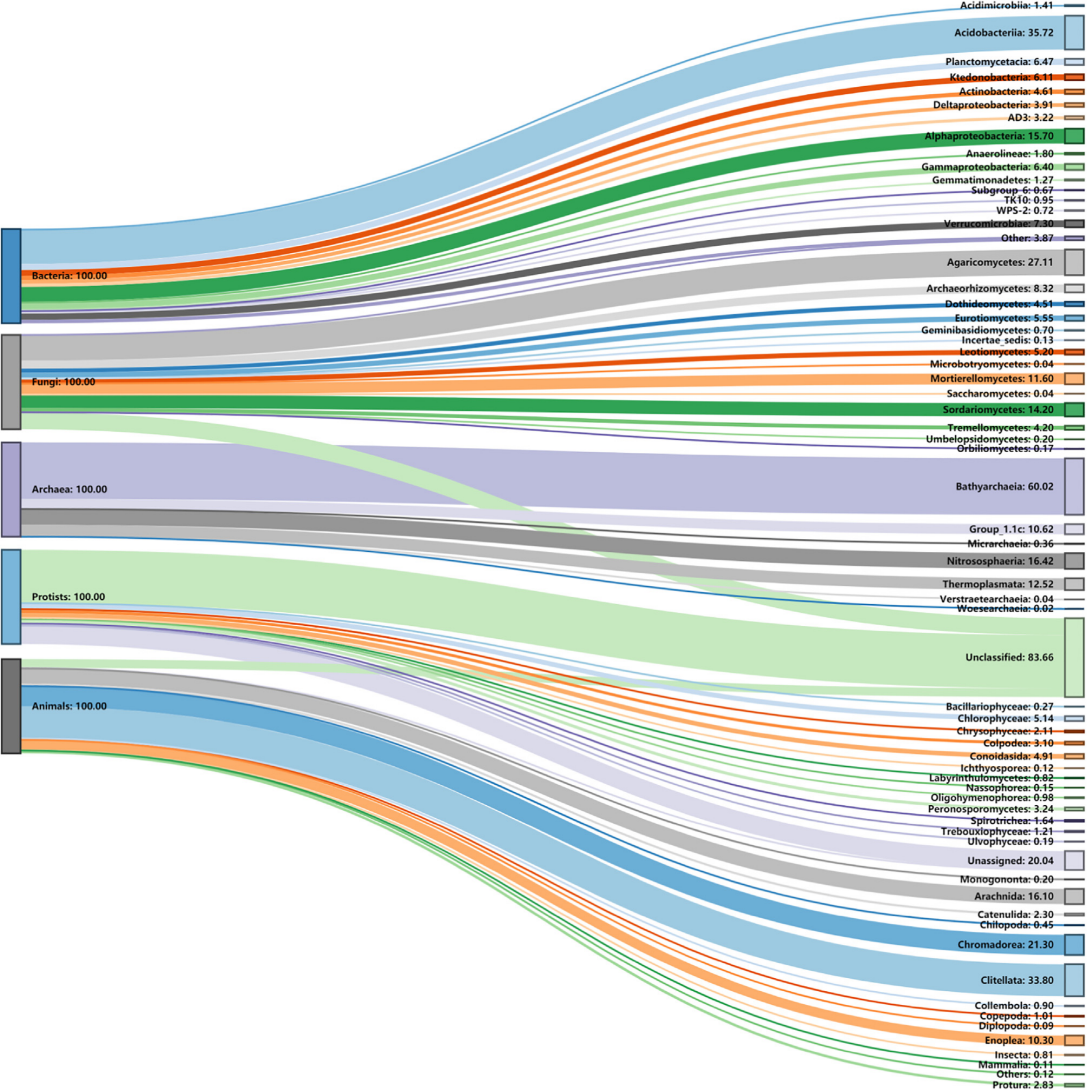
Sankey diagram of relative abundance (%) of top 15 classes for soil microbial (Bacteria, Fungi, Archaea, Protists) and animal communities. The figure was created using the online website http://sankeymatic.com/build/. The vector code (SVG) for this figure is available in supplementary materials.

**Fig. 2:**
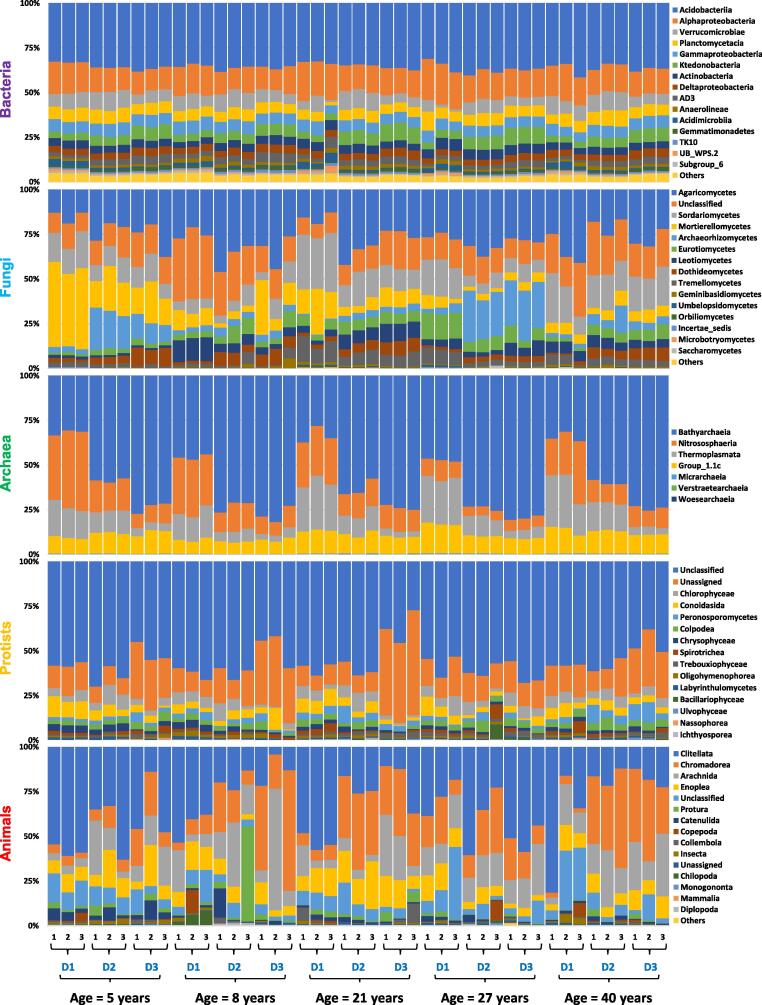
Relative abundance (%) of top 15 soil microbial (Bacteria, Fungi, Archaea, Protists) and animal communities for all samples. Here, 1,2,3 are for replication 1, replication 2 and replication 3, while, D1, D2, D3 are about different soil depths i.e., D1 (0–10 cm), D2 (10–20 cm) and D3 (20–40 cm).

**Fig. 3:**
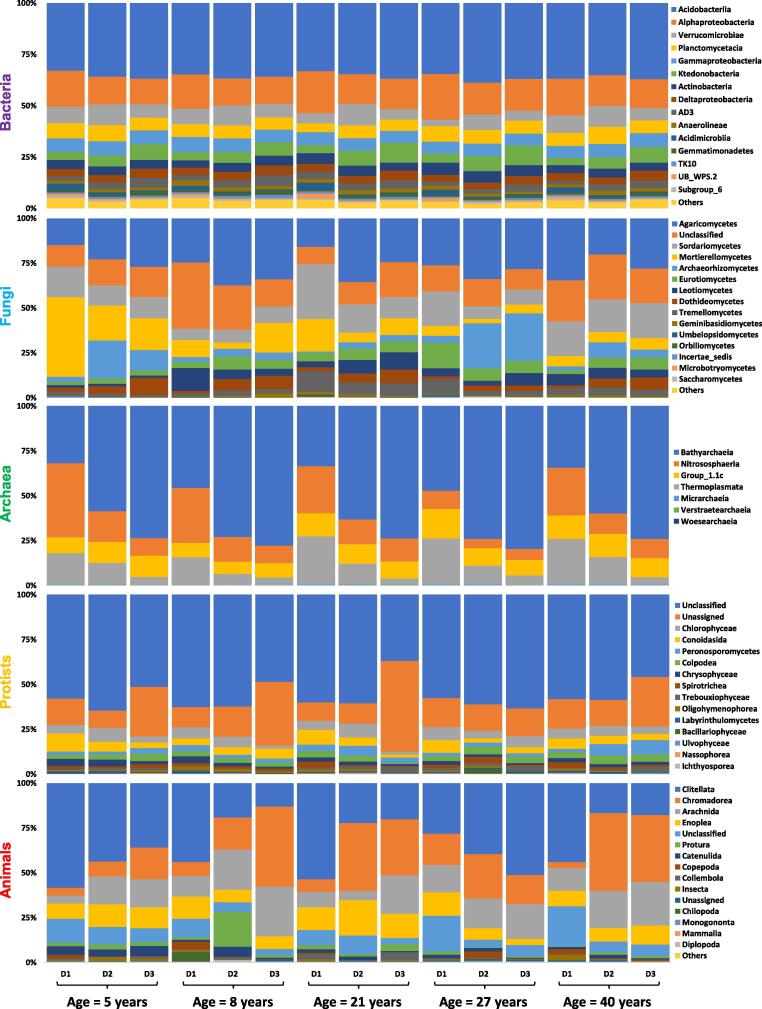
Combined relative abundance (%) of top 15 soil microbial (Bacteria, Fungi, Archaea, Protists) and animal communities for all samples after pooling the replications. Here, D1, D2, D3 are about different soil depths i.e., D1 (0–10 cm), D2 (10–20 cm) and D3 (20–40 cm).

Significant decreasing and increasing trends for the relative abundance of microbial and animal communities (top 15 classes) were observed with respect to changes in stand age and soil depth profiles (Figs. 2,3; Supplementary Fig S2,3; Supplementary Table 1). Briefly, among bacteria, Acidobacteriia were the most abundant class i.e., 36%, followed by Alphaproteobacteria (16%) and Verrucomicrobiae (7%) (Fig. 1). Relative abundance of Acidobacteriia increased with the increase in depth however an opposite trend was observed for the Alphaproteobacteria as their abundance reduced with the increase in depth symmetrically. Interestingly, Verrucomicrobiae showed increased abundance at 10–20 cm soil depth for all the stand ages. With an increase in stand age, the relative abundance of Alphaproteobacteria increased upto 27 years significantly (*p* = 0.05), while Verrucomicrobiae showed a significant decrease (*p* = 0.05) (Supplementary Fig S3a; Supplementary Table 1). Agaricomycetes were the most abundant (27%) class among fungi, followed by Sordariomycetes (14%) and Mortierellomycetes (12%) (Fig. 1). A contrasting trend was observed for all the fungal classes with the response to increasing age and soil depth. However, Sordariomycetes abundance was recorded relatively high at 0–10 cm soil depth and for 21 and 40 years stand age (*p* = 0.001), while Mortierellomycetes were found significantly abundant at the early stage of plantation (*p* < 0.001) i.e., 5 years age (Supplementary Fig S3b; Supplementary Table 1). On a similar note, dominant Archaea classes were Bathyarchaeia (60%), Nitrososphaeria (16%) and Thermoplasmata (13%) (Fig. 1). Bathyarchaeia exhibited an increasing trend with respect to an increase in soil depth, while contrastingly, both Nitrososphaeria and Thermoplasmata reduced with the increasing soil depth (Supplementary Fig S3c). Interestingly, Nitrososphaeria significantly reduced with the increase in stand age up to 27 years (*p* = 0.05), while Thermoplasmata increased with an increase in stand age from 8 to 20 years (Supplementary Fig S3c; Supplementary Table 1). Most of the sequences for protists were unclassified (56%) and unassigned (19%) at the class level (Fig. 1). Among the classified protists classes, Chlorophyceae, Conoidasida and Peronosporomycetes were dominant, exhibiting 5.1%, 4.8% and 3.2% relative abundance respectively (Fig. 1). Conoidasida significantly reduced with the increase in soil depth (*p* < 0.001) while Peronosporomycetes showed an opposing trend. Chlorophyceae showed inconsistent trends with a change in soil depth profiles (*p* < 0.001) (Supplementary Fig S3d; Supplementary Table 1). Interestingly, a significant high relative abundance of Peronosporomycetes was observed in 40 years age stands (*p* = 0.001). Among animal communities, Clitellata (34%) were the most dominant class, while Chromadorea (21%) and Arachnida (16%) were also among the dominant classes (Fig. 1). For most of the stand ages of Chinese fir plantation, Clitellata abundance reduced with the increase in soil depth (*p* = 0.001), while Arachnida (*p* = 0.05) and Chromadorea (*p* < 0.001) followed an opposite trend (Supplementary Fig S3e; Supplementary Table 1).

**Fig. 4:**
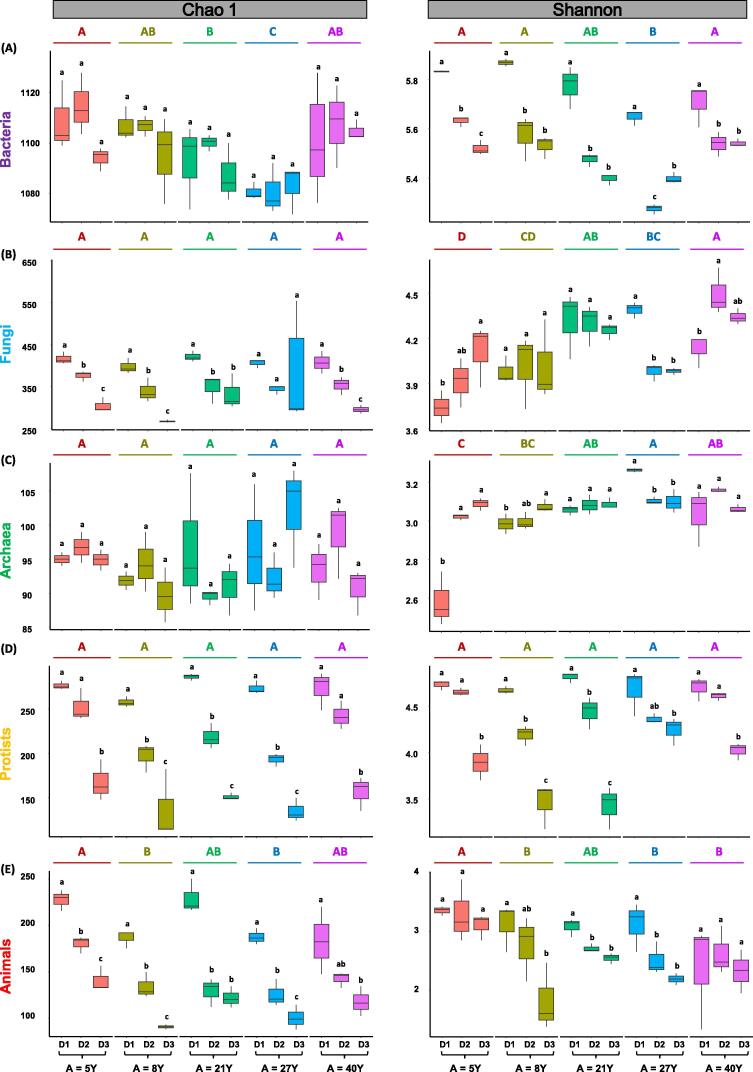
Box plots for alpha diversity (Chao 1 and Shannon indices) of soil microbial (Bacteria, Fungi, Archaea, Protists) and animal communities. Here, D1, D2, D3 are about different soil depths i.e., D1 (0–10 cm), D2 (10–20 cm) and D3 (20–40 cm). K). Error bars in the boxplots with different lowercase letters show significant differences between different depths, while different uppercase letters describe significant differences among stand ages (LSD test).

Alpha diversity of microbial (Bacteria, Fungi, Archaea, Protists) and animal communities was estimated by Chao1 and Shannon indices. Significant trends among alpha diversity of all the biotic communities were observed with changes in stand age and soil depth profiles (Fig. 4; Supplementary Fig S4). For example, on average, bacterial alpha diversity decreased significantly up to the age of 27, followed by an increase at 40 years stand in both Chao1 (*p =* 0.001) and Shannon indices (*p <* 0.001). However, Shannon indices indicated a significant decrease in bacterial alpha diversity with the increase in soil depth (*p <* 0.001) (Fig. 4-A). A similar trend was recorded in fungal diversity by Chao1 indices (*p <* 0.001) (Fig. 4-B), while archaea exhibited a contrasting significant opposite trend (Shannon indices; *p <* 0.001) (Fig. 4-C). For protist (Fig. 4-D) and animal (Fig. 4-E) communities, a significant decreasing trend was observed with an increase in soil depth by Chao 1 and Shannon indices. Other than the bacterial communities, contrasting significant trends for the alpha diversity of all the other communities were recorded with an increase in stand ages (Chao 1 and Shannon indices) (Fig. 4; Supplementary Fig S4).

**Fig. 5:**
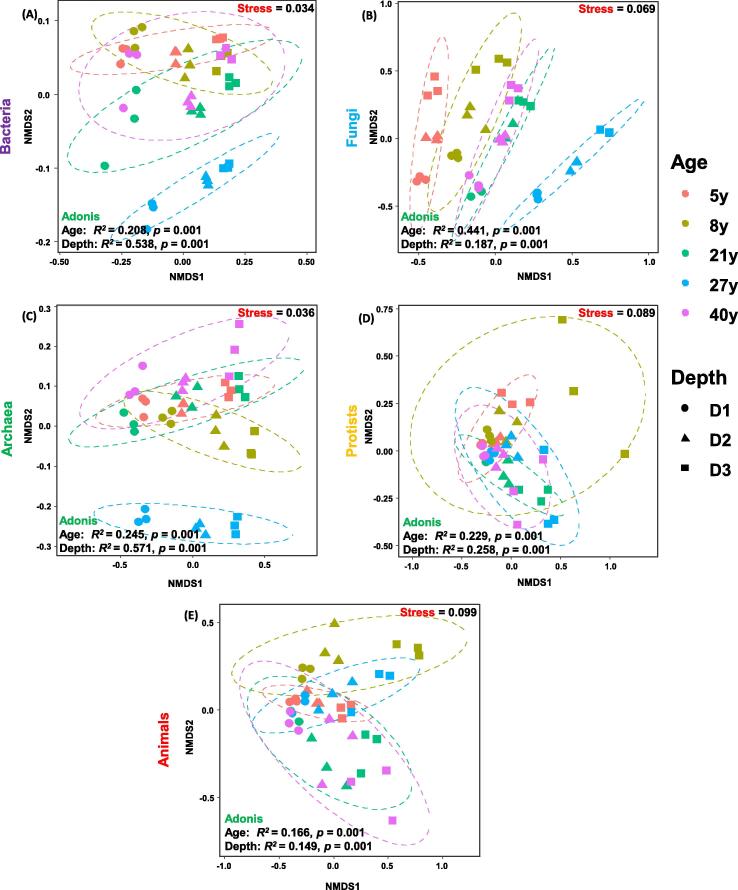
Plots of the non-metric dimensional scaling ordination for soil microbial (Bacteria, Fungi, Archaea, Protists) and animal communities. Here, stand ages (5, 8, 21, 27, 40 years) have been designated with different colors while soil depths, D1 (0–10 cm), D2 (10–20 cm) and D3 (20–40 cm) are represented with different shapes. Stress values, p-values and ANOSIM & Adonis test confirmatory values are indicated in the figure.

Non-metric multidimensional scaling (NMDS) analysis revealed that soil samples from different soil depths and stand ages formed distinct and overlapping clusters in ordination space (Fig. 5) with significant difference (ANOSIM & Adonis test; *p* = 0.001) (Supplementary Table 2). We observed significant differences in microbial and animal communities with respect to soil depth and stand age. These differences were larger for soil archaea (Age-*R^2^ =* 0.245, Depth- *R^2^ =* 0.571), bacterial (Age-*R^2^ =* 0.208, Depth- *R^2^ =* 0.538) and fungal communities (Age-*R^2^ =* 0.441, Depth- *R^2^ =* 0.187), followed by protists (Age-*R^2^ =* 0.229, Depth- *R^2^ =* 0.258) and animals (Age-*R^2^ =* 0.166, Depth- *R^2^ =* 0.149), which indicates that the soil fungal, bacterial and archaea communities were more influenced by stand age and soil depth (Fig. 5A-E; Supplementary Table 2). Other than the soil fungi and animal communities, all other microbial communities were more sensitive to soil depth as compared to stand age (Adonis Test) (Supplementary Table 1).

### Potential drivers of soil microbial and animal community diversity

**Fig. 6:**
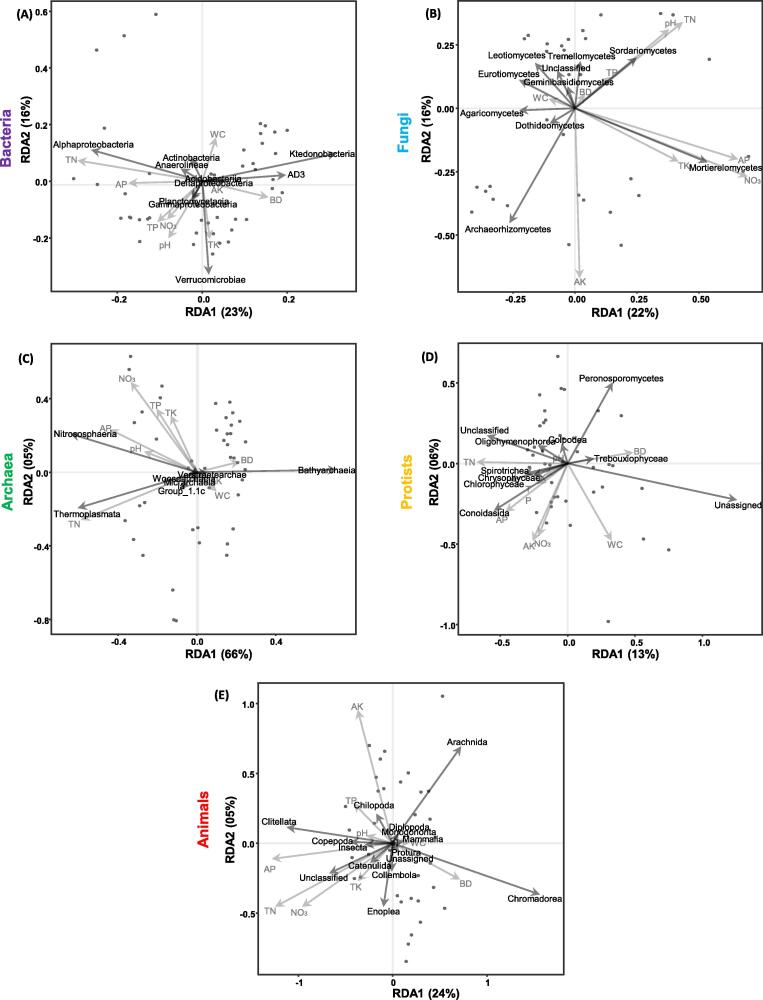
Redundancy analysis (RDA) illustrating the effects of environmental factors (arrows) on soil microbial (Bacteria, Fungi, Archaea, Protists) and animal community’s structure (dot symbols) across all soil samples. The values of axes 1 and 2 are the percentage that can be explained by the corresponding axis.

To understand the main drivers for the diversity of microbial and animal communities, we tried to find a correlation between soil physiochemical properties and soil biotic communities through Redundancy analysis (RDA) (Fig. 6; Supplementary Table 3) and creating a heatmap through Pearson correlation (Supplementary Fig S5). The abundance of microbial and animal communities’ classes was variable and differently coordinated with soil physiochemical properties. For example, TC and TN were the most significant influencing factors (*p <* 0.001) for all the communities with variable p-values followed by pH, TP, AP, NO_3_ and AK. We found collinearity among TC, TN and C/N-Ratio on the basis of VIF. Therefore, we selected TN in the final model (Supplementary Table 3). For bacteria, TN was predicted to have a significant influence on Alphaproteobacteria (*p <* 0.001). We also observed a significant positive correlation among Gammaproteobacteria and Planctomycetacia with pH (*p =* 0.001), TP (*p =* 0.01) and NO_3_ (*p =* 0.05) (Fig. 6-A). For fungi, TN (*p =* 0.001), pH (*p =* 0.001), NO_3_ (*p =* 0.001) and AP (*p <* 0.001) exhibited a significant positive correlation with Sordariomycetes and Mortierellomycetes (Fig. 6-B). Thermoplasmata had a significant positive influence on TN (*p <* 0.001) while Nitrososphaeria was predicted to have been associated with changes in AP (*p =* 0.01) and pH (*p <* 0.001) (Fig. 6-C). For protists, RDA predicted that Spirotichea had a positive correlation with TN (*p <* 0.001) (Fig. 6-D), while for animals, TN was influenced by the majority of unassigned animal classes (*p <* 0.001) (Fig. 6-E).

## Discussion

Soil biotic communities are vital ecological indicators for forest ecosystems. Different forest management and rotation practices continuously change soil biotic communities’ structure, composition, diversity and correlations with soil nutrients, impacting plant growth and metabolism. In this study, we utilized high-throughput sequencing to unveil the diversity of soil biotic communities amongst the chronosequence of Chinese fir across different vertical soil profiles. The results revealed both expected and novel trends for abundance, diversity and dynamic relationships between soil organisms and environmental drivers.

Soil physiochemical properties exhibited notable contrasting trends across different stand ages and soil depth profiles. We observed that key soil physiochemical factors, such as TC, TN, TP, AP, pH and NO_3_ decreased with the increase in soil depth (0–40 cm) (Supplementary Fig S1). It is verified that TC [44], TN and TP [45] decrease consistently with increasing soil depth (0–60 cm) across Chinese fir chronosequence. In acidic soils, AP and pH are considered closely related factors showing similar trends, as it has already been reported that soil pH constantly decreased with stand age across Chinese fir plantations, resulting in AP decrease because the low pH in acid soils drives phosphate to bind to Fe and Al precipitates [46], [47]. The decrease in NO_3_ is correlated with irrigation because continuous and heavy rainfall results in the leaching of NO_3_ [48]. We observed variable patterns for TK, AK, BD, consistent with the findings of Selvalakshmi et al. [49] and Chen et al. [44], respectively. For most of the soil nutrients (TN, TP, TK, AP, Ak) (Supplementary Fig S1), we observed variable increase/decrease trends with respect to stand age, confirming that the stand age played a minor role in changing the status of most of the soil nutrients [44]. However, some soil nutrients (TC, pH, NO_3_) exhibited changes with stand age. For example, up to the age 27, TC showed an increase followed by a slightly decreasing trend at 40 years stands. On the contrary, soil pH and NO_3_ exhibited a significant opposite trend. These results are in line with the findings of Chen et al. [44] and Zhang et al. [50].

Illumina Hiseq2500 sequencing revealed variable significant increase/decrease trends among the top 15 classes of soil biotic communities with respect to changes in stand age and soil depth profiles. For bacterial classes, Acidobacteriia, alphaproteobacteria and verrucomicrobiae were the most abundant bacterial classes among all the stand ages and soil profiles. These findings are consistent with the previous reports [8], [15]. Moreover, it is established that the high dominance of Acidobacteriia (36%) (Fig. 1) is due to the soil acidity (4 < pH < 5)[51], which was in good agreement with our findings. We found that the relative abundance of Acidobacteriia increased with the increase in soil depth however, an opposite trend was observed for the alphaproteobacteria (Supplementary Fig S3a). A similar shift in bacterial community composition was observed along with deep soil profiles in monospecific and mixed stands of *Eucalyptus grandis* and *Acacia mangium* [52]. Different Chinese fir stand ages posed significant narrow variations in the relative abundance of major bacterial classes [8]. These observations are connected with the narrow range of pH change (approximately 0.3 unit) detected among different stand ages in our findings (Supplementary Fig S1-F), which is supported by earlier research that demonstrated pH as the best predictor for changes in bacterial community structure and composition by surveying 88 soils across America [51]. As reported by Liu et al. [8] and Buée et al. [53], we recorded high abundance of Agaricomycetes (27%), phylum Basidiomycota (Fig. 1). These fungi are considered lignin decomposers, and therefore, with the increase in forest age, the litters are increased, leading to the rapid growth and abundance of Basidiomycota (Agaricomycetes)[54]. Stand age and soil depth profiles did not exhibit any particular trend among the major classes of fungi; however, we observed a relatively high abundance of Sordariomycetes in 0–10 cm deep soil samples (Supplementary Fig S3b) that was already a confirmed finding [54]. A comparatively high dominance was observed for Mortierellomycetes in 5 years age stands of Chinese fir (Supplementary Fig S3b), which is perhaps due to the capability of this particular fungi to decompose the limbs of plants and animals remains of the previous plantation after the rotation [55], and to support root development during initial years of tree growth through phosphorus transformation from an insoluble to a soluble form that can then be directly utilized by plants [56]. Our findings revealed Bathyarchaeia (60%) as the most abundant archaeal class, belonging to the phylum Crenarchaeota (Fig. 1). According to our brief knowledge, there are no previous reports regarding the abundance of archaea, protists and animals with respect to various soil profiles in Chinese fir chronosequence. However, these archaea are found abundantly in soils that have been subjected to strong ecological and management treatments (clear-cutting and burning)[57], which is very common in Chinese fir plantations [2]. Few reports disclosed that archaeal dominance is related to ammonium oxidization at Alaska and Qinghai-Tibetan forest sites [58], [59]. Increasing soil depth showed an increase in Bathyarchaeia, as supported by an early report [58], while Nitrososphaeria (Thaumarchaeota) and Thermoplasmata (Euryarchaeota) exhibited reduction with the increasing soil depth (Supplementary Fig S3c). In rhizospheres, Thaumarchaeota exist as ammonia-oxidizers, playing vital roles in biogeochemical cycles (nitrogen & carbon cycle)[60], while Euryarchaeota seems to be directly proportional to the existence of mycorrhizal fungi [61].

Protists are overlooked and are not well studied, but we believe that all the soil biotic communities play some of the other role in balancing the soil ecosystem. Our results exhibited unclassified (56%) and unassigned (19%) protist classes (Fig. 1), clearly indicating that they have been poorly understood and more research is required to classify them at various taxonomic levels. Among other classified protists, Chlorophyceae (phylum Chlorophyta) were found most abundant (5.1%) (Fig. 1). Some previous reports related to biological soil crusts [62] and forest forming tree species suggest the higher abundance of Chlorophyta in soil [63]. In our findings, Conoidasida (phylum Apicomplexa) reduced while Peronosporomycetes (Clade Rhizaria) increased with an increase in soil depth (Supplementary Fig S3d). Not much information is available about the Conoidasida or Peronosporomycetes regarding their trend with the changes in stand age and vertical soil depth profiles in literature. However, some researchers observed the spacio-temporal structure of Rhizaria for temperate agriculture and grasslands ecosystem [64], [65]. Among animal communities, we recorded the higher relative abundance of Clitellata (34%) belonging to phylum Annelida (Fig. 1). Not many reports exist in the literature about the abundance and diversity of soil animal communities in the forest ecosystem. But it is reported that the Annelida population is relatively higher in woody savannahs as compared to pastures, grasslands and fields [66]. We observed that for most of the stand ages, Clitellata abundance reduced while Arachnida and Chromodorea increased with increasing soil depth (Supplementary Fig S3e). In our understanding, mostly the members of Arachnida are spiders that don’t like the places with greater human activity and management practices that may lead them to stay at increasing soil depth (40 cm) or even more [67]. Similarly, Chromodorea are Nematodes whose population is correlated with the soil moisture content in the forest ecosystem [68]. The dynamic shift and trends among nematodes may be linked to the trend of WC in our study. Members of Clitellata (phylum Annelida) are earthworms that burrow in the soil or live near the surface, generally in moist leaf litter [69]. These are very important contributors to soil fertility as they loosen the soil by burrowing and help ion mixing the organic and mineral matter through acceleration in the decomposition process [70].

Our results explained that the stand age impacted the alpha diversity of bacteria showing a significant decrease and increasing trend. However, a significant decreasing trend in alpha diversity was observed with an increase in soil depth for all the biotic communities except archaea that showed a contrasting opposite trend (Fig. 4; Supplementary Fig S4). For example, on average, bacterial alpha diversity decreased up to the age of 27 significantly, followed by an increase at 40 years stand, which is directly proportional to the changes in soil pH. Significant correlations have been frequently found between the pH and bacterial alpha-diversity in different soils [71], [72] and lake sediments [73], indicating that pH is a predictor for soil microbial diversity. This is recently confirmed by a global *meta*-analysis, stating that soil pH is the top most indicator for changes in microbial diversity [74]. The decreasing trend in fungal [14], protists [75] and animal [11] communities diversity, while the opposite contrasting trend in archaea communities was observed across reforested, agricultural and temperate ecosystems [11]. Protist communities are considered the most diverse and dominating in neotropical forests [76] and are influenced by changes in soil nitrogen [75]. However, in the tropical forest ecosystem, our findings are novel with respect to different soil depths and stand ages that may also be correlated with nitrogen availability in soil, but we believe that more specific research is required to understand this dynamic aspect. Generally, regarding richness, Protist and animals follow a similar trend like bacteria and fungi while archaea exhibit an opposite trend [11], which is similar to our findings as well (Chao1 index). As mentioned earlier, Animal diversity is also related to TC and least disturbance and tillage activities [77].

Among all the soil biotic communities, we found that the beta diversity of fungal communities was more influenced by stand age (Adonis test) (Fig. 5), previously confirmed by Lu et al. [17]. Our results explained that TN was the most significant influencing factor for all the communities, followed by TP, pH and NO_3_. TN correlation with bacterial, fungal [8], [78], archaeal [79] and animal communities [80]; pH, TP and NO_3_ correlation with Proteobacteria [14], [51] has been previously documented.

## Conclusions

Using various soil vertical profiles to study the assemblage of soil biotic communities across the chronosequence of Chinese fir plantations, we find perhaps the most comprehensive evidence showing the complexity of belowground ecology. Our results showed that with the increase in soil depth, soil biotic communities exhibited a decreasing trend in alpha diversity of bacteria, fungi, protists and animals; however, archaea showed an opposite contrasting trend. The most abundant soil bacterial, fungal, archaeal, protist and animal classes were Acidobacteriia, Agaricomycetes, Bathyarchaeia, Chlorophyceae and Clitellata, respectively. Correlation of vertical distribution of biotic communities and variations in soil physiochemical properties explained that TN, AP and soil pH were the most influencing factors for changes in soil biotic communities. Our results highlighted that microbial and animal richness is strongly influenced by soil properties in a near-uniform manner thus explaining the importance of the dynamics between biotic and abiotic processes that drive the organization of belowground biological diversity. Soil can be regarded as “ecological black boxes” as it provides differential insights among belowground micro and macro-biota. Therefore, we believe that this study represents a step toward an insightful understanding of all the soil biotic communities in continuous plantations of Chinese fir. We believe that our study will facilitate in better understanding not only the major soil communities (Bacteria and Fungi) but also the other important communities (Archaea, Protists, Animals) diversity and structure in response to different stand ages and vertical soil profiles. It will serve as a theoretical base for developing more sustainable management practices in the forest ecosystem.

**Declarations**

Ethics approval and consent to participate.

Not applicable.

**Consent for publication**

Not applicable.

**Availability of data and material**

The datasets generated and/or analyzed during the current study are available at www.datadryad.org. via https://doi.org/10.5061/dryad.4mw6m908r. The data will be made public upon acceptance of this article. The data temporary link for peer review is as under. https://datadryad.org/stash/share/XRRmPBR9nneeDHhi2YtMxfVH0Hkw6Dq0hly0oxy6S6w.

**Credit Authorship Statement**

HYHC and ZH designed the experiment. WI conducted the sampling, analysis, written and finalized the manuscript. HSAS, MA and MT helped in software statistical analysis. ZW helped in analyzing soil physiochemical properties. ZH and HYCH supervised and provided funding for the project.

## Declaration of Competing Interest

The authors declare that they have no known competing financial interests or personal relationships that could have appeared to influence the work reported in this paper.

## References

[1] Chen G, Yang Y, Yang Z, Xie J, Guo J, Gao R, et al. Accelerated soil carbon turnover under tree plantations limits soil carbon storage. Scientific Reports 2016;6. doi:10.1038/srep19693. 10.1038/srep19693 PMC4726314 26805949

[2] Farooq T.H., Yan W., Rashid M.H.U., Tigabu M., Gilani M.M., Zou X.H. (2019). Chinese fir (Cunninghamia Lanceolata) a green gold of China with continues decline in its productivity over the successive rotations: A review. Appl Ecol Environ Res.

[3] Kooch Y., Rostayee F., Hosseini S.M. (2016). Effects of tree species on topsoil properties and nitrogen cycling in natural forest and tree plantations of northern Iran. Catena.

[4] Farooq TH, Gautam NP, Rashid MH., Gilani MM, Nemin W, Nawaz MF, et al. Contributions of Agroforestry on Socio-economic Conditions of Farmers in Central Punjab, Pakistan – A Case Study. Cercetari Agronomice in Moldova 2018;51:91–101. doi:10.2478/cerce-2018-0020.

[5] Selvaraj S., Duraisamy V., Huang Z., Guo F., Ma X. (2017). Influence of long-term successive rotations and stand age of Chinese fir (Cunninghamia lanceolata) plantations on soil properties. Geoderma.

[6] Zhijun H., Selvalakshmi S., Vasu D., Liu Q., Cheng H., Guo F. (2018). Identification of indicators for evaluating and monitoring the effects of Chinese fir monoculture plantations on soil quality. Ecol Ind.

[7] Lin C., Yang Y., Guo J., Xie J., Zhao Y. (2010). Decomposition dynamics and nutrient release of mixed fineroots of Castanopsis carlesii and Cunninghamia lanceolata. Shengtai Xuebao/ Acta Ecologica Sinica.

[8] Liu X, Wang Y, Liu Y, Chen H, Hu Y. Response of Bacterial and Fungal Soil Communities to Chinese Fir (Cunninghamia lanceolate) Long-Term Monoculture Plantations. Frontiers in Microbiology 2020;11. doi:10.3389/fmicb.2020.00181. 10.3389/fmicb.2020.00181 PMC7058989 32184765

[9] Islam W., Noman A., Naveed H., Huang Z., Chen H.Y.H. (2020). Role of environmental factors in shaping the soil microbiome. Environ Sci Pollut Res.

[10] Wei L.L., Lu C.Y., Ding J., Yu S. (2016). Functional relationships between arbuscular mycorrhizal symbionts and nutrient dynamics in plant-soil-microbe system. Shengtai Xuebao/ Acta Ecologica Sinica.

[11] George PBL, Lallias D, Creer S, Seaton FM, Kenny JG, Eccles RM, et al. Divergent national-scale trends of microbial and animal biodiversity revealed across diverse temperate soil ecosystems. Nature Communications 2019;10. doi:10.1038/s41467-019-09031-1. 10.1038/s41467-019-09031-1 PMC6405921 30846683

[12] Goldmann K, Schöning I, Buscot F, Wubet T. Forest management type influences diversity and community composition of soil fungi across temperate forest ecosystems. Frontiers in Microbiology 2015;6. doi:10.3389/fmicb.2015.01300. 10.3389/fmicb.2015.01300 PMC4656839 26635766

[13] Dieng A., Ndoye I., Duponnois R., Baudoin E. (2014). Effects of Jatopha curcas L. plantation on soil bacterial and fungal communities. Soil Biol Biochem.

[14] Jiao S, Chen W, Wang J, Du N, Li Q, Wei G. Soil microbiomes with distinct assemblies through vertical soil profiles drive the cycling of multiple nutrients in reforested ecosystems. Microbiome 2018;6. doi:10.1186/s40168-018-0526-0. 10.1186/s40168-018-0526-0 PMC6104017 30131068

[15] Wang Q, Wang C, Yu WW, Turak A, Chen D, Huang Y, et al. Effects of nitrogen and phosphorus inputs on soil bacterial abundance, diversity, and community composition in chinese fir plantations. Frontiers in Microbiology 2018;9. doi:10.3389/fmicb.2018.01543. 10.3389/fmicb.2018.01543 PMC6060263 30072961

[16] Ochoa-Hueso R, Arca V, Delgado-Baquerizo M, Hamonts K, Piñeiro J, Serrano-Grijalva L, et al. Links between soil microbial communities, functioning, and plant nutrition under altered rainfall in Australian grassland. Ecological Monographs 2020;90. doi:10.1002/ecm.1424.

[17] Lu N, Xu X, Wang P, Zhang P, Ji B, Wang X. Succession in arbuscular mycorrhizal fungi can be attributed to a chronosequence of Cunninghamia lanceolata. Scientific Reports 2019;9. doi:10.1038/s41598-019-54452-z. 10.1038/s41598-019-54452-z PMC6889488 31792242

[18] Anderson L.J. (2011). Aboveground-belowground linkages: Biotic interactions, ecosystem processes, and global change. Eos, Transactions American Geophysical Union.

[19] Urbanová M., Šnajdr J., Baldrian P. (2015). Composition of fungal and bacterial communities in forest litter and soil is largely determined by dominant trees. Soil Biol Biochem.

[20] IUSS Working Group WRB (2014). World reference base for soil resources 2014. International soil classification system for naming soils and creating legends for soil maps..

[21] Johnson E.A., Miyanishi K. (2008). Testing the assumptions of chronosequences in succession. Ecol Lett.

[22] Walker L.R., Wardle D.A., Bardgett R.D., Clarkson B.D. (2010). The use of chronosequences in studies of ecological succession and soil development. J Ecol.

[23] Chen H.YH., Popadiouk R.V. (2002). Dynamics of North American boreal mixedwoods. Environmental Reviews.

[24] Pansu M., Gautheyrou J. (2006). Handbook of soil analysis: Mineralogical, organic and inorganic methods. Springer Science & Business. Media.

[25] Sagova-Mareckova M., Cermak L., Novotna J., Plhackova K., Forstova J., Kopecky J. (2008). Innovative methods for soil DNA purification tested in soils with widely differing characteristics. Appl Environ Microbiol.

[26] Caporaso J.G., Lauber C.L., Walters W.A., Berg-Lyons D., Lozupone C.A., Turnbaugh P.J. (2011). Global patterns of 16S rRNA diversity at a depth of millions of sequences per sample. PNAS.

[27] Epp LS, Boessenkool S, Bellemain EP, Haile J, Esposito A, Riaz T, et al. New environmental metabarcodes for analysing soil DNA: Potential for studying past and present ecosystems. Molecular Ecology 2012;21:1821–33. doi:10.1111/j.1365-294X.2012.05537.x. 10.1111/j.1365-294X.2012.05537.x 22486821

[28] Behnke A., Engel M., Christen R., Nebel M., Klein R.R., Stoeck T. (2011). Depicting more accurate pictures of protistan community complexity using pyrosequencing of hypervariable SSU rRNA gene regions. Environ Microbiol.

[29] Lee P.Y., Costumbrado J., Hsu C.Y., Kim Y.H. (2012). Agarose gel electrophoresis for the separation of DNA fragments. J. Visualized Experiments.

[30] Edgar R.C., Haas B.J., Clemente J.C., Quince C., Knight R. (2011). UCHIME improves sensitivity and speed of chimera detection. Bioinformatics.

[31] Edgar R.C. (2013). UPARSE: Highly accurate OTU sequences from microbial amplicon reads. Nat Methods.

[32] Kõljalg U., Nilsson R.H., Abarenkov K., Tedersoo L., Taylor A.F.S., Bahram M. (2013). Towards a unified paradigm for sequence-based identification of fungi. Mol Ecol.

[33] Quast C, Pruesse E, Yilmaz P, Gerken J, Schweer T, Yarza P, et al. The SILVA ribosomal RNA gene database project: Improved data processing and web-based tools. Nucleic Acids Research 2013;41. doi:10.1093/nar/gks1219. 10.1093/nar/gks1219 PMC3531112 23193283

[34] Sugawara E., Nikaido H. (2014). Properties of AdeABC and AdeIJK efflux systems of Acinetobacter baumannii compared with those of the AcrAB-TolC system of Escherichia coli. Antimicrob Agents Chemother.

[35] McMurdie PJ, Holmes S. Phyloseq: An R Package for Reproducible Interactive Analysis and Graphics of Microbiome Census Data. PLoS ONE 2013;8. doi:10.1371/journal.pone.0061217. 10.1371/journal.pone.0061217 PMC3632530 23630581

[36] Ssekagiri A, T. Sloan W, Zeeshan Ijaz U. microbiomeSeq: An R package for analysis of microbial communities in an environmental context. ISCB Africa ASBCB Conference 2017.

[37] Oksanen J., Blanchet F.G., Kindt R., Legendre P., Minchin P.R., O’Hara R.B. (2013). Vegan: Community Ecology Package. R package version 2.0-9. Community Ecology Package.

[38] Benjamini Yoav, Hochberg Yosef (1995). Controlling the False Discovery Rate: A Practical and Powerful Approach to Multiple Testing. J Roy Stat Soc: Ser B (Methodol).

[39] Torondel Belen, Ensink Jeroen H.J., Gundogdu Ozan, Ijaz Umer Zeeshan, Parkhill Julian, Abdelahi Faraji (2016). Assessment of the influence of intrinsic environmental and geographical factors on the bacterial ecology of pit latrines. Microb Biotechnol.

[40] Bollinger Galen, Belsley David A., Kuh Edwin, Welsch Roy E. (1981). Regression Diagnostics: Identifying Influential Data and Sources of Collinearity. J Mark Res.

[41] Legendre P., Oksanen J., ter Braak C.J.F. (2011). Testing the significance of canonical axes in redundancy analysis. Methods Ecol Evol.

[42] Warnes G.R., Bolker B., Bonebakker L., Gentleman R., Huber W., Liaw A. (2015). Various R programming tools for plotting data. The Comprehensive R Archive Network (CRAN).

[43] Wickman H, François R, Henry L, Muller K. dlpyr: A Grammar of Data Manipulation. CRAN Repository 2021.

[44] Chen Guang-Shui, Yang Zhi-Jie, Gao Ren, Xie Jin-Sheng, Guo Jian-Fen, Huang Zhi-Qun (2013). Carbon storage in a chronosequence of Chinese fir plantations in southern China. For Ecol Manage.

[45] Wu H, Xiang W, Ouyang S, Xiao W, Li S, Chen L, et al. Tree growth rate and soil nutrient status determine the shift in nutrient-use strategy of Chinese fir plantations along a chronosequence. Forest Ecology and Management 2020;460. doi:10.1016/j.foreco.2020.117896.

[46] Yan Tao, Lü Xiao-Tao, Zhu Jiao-Jun, Yang Kai, Yu Li-Zhong, Gao Tian (2018). Changes in nitrogen and phosphorus cycling suggest a transition to phosphorus limitation with the stand development of larch plantations. Plant Soil.

[47] Wu Huili, Xiang Wenhua, Chen Liang, Ouyang Shuai, Xiao Wenfa, Li Shenggong (2020). Soil Phosphorus Bioavailability and Recycling Increased with Stand Age in Chinese Fir Plantations. Ecosystems.

[48] Bahmani Omid, Sabziparvar Ali Akbar, Javadi Hossein, Pak Vahid Atlassi, Boroomand Nasab Saeed (2019). Evaluation of Soil Nitrate Accumulation under Different Fertigation Regimes and Simulation by the Hydrus-1D Model. Water Conservation Science and Engineering.

[49] Selvalakshmi S, Vasu D, Zhijun H, Guo F, Ma XQ. Soil nutrients dynamics in broadleaved forest and Chinese fir plantations in subtropical forests. Journal of Tropical Forest Science 2018;30:242–51. doi:10.26525/jtfs2018.30.2.242251.

[50] Zhang Xie, Wu Zhenming, Xu Zhongkun, Xu Liang, Xu Qingqian, Lin Jianzhong (2021). Estimated biomass carbon in thinned Cunninghamia lanceolate plantations at different stand-ages. J For Res.

[51] Lauber Christian L., Hamady Micah, Knight Rob, Fierer Noah (2009). Pyrosequencing-based assessment of soil pH as a predictor of soil bacterial community structure at the continental scale. Appl Environ Microbiol.

[52] Pereira Arthur Prudêncio de Araujo, Andrade Pedro Avelino Maia de, Bini Daniel, Durrer Ademir, Robin Agnès, Bouillet Jean Pierre (2017). Shifts in the bacterial community composition along deep soil profiles in monospecific and mixed stands of Eucalyptus grandis and Acacia mangium. PLoS ONE.

[53] Buée M, Reich M, Murat C, Morin E, Nilsson RH, Uroz S, et al. 454 Pyrosequencing analyses of forest soils reveal an unexpectedly high fungal diversity. New Phytologist 2009;184:449–56. doi:10.1111/j.1469-8137.2009.03003.x. 10.1111/j.1469-8137.2009.03003.x 19703112

[54] Ko D, Yoo G, Yun ST, Jun SC, Chung H. Bacterial and fungal community composition across the soil depth profiles in a fallow field. Journal of Ecology and Environment 2017;41. doi:10.1186/s41610-017-0053-0.

[55] Steidinger BS, Crowther TW, Liang J, Van Nuland ME, Werner GDA, Reich PB, et al. Climatic controls of decomposition drive the global biogeography of forest-tree symbioses. Nature 2019;569:404–8. doi:10.1038/s41586-019-1128-0. 10.1038/s41586-019-1128-0 31092941

[56] Zhang T, Wang Z, Lv X, Li Y, Zhuang L. High-throughput sequencing reveals the diversity and community structure of rhizosphere fungi of Ferula Sinkiangensis at different soil depths. Scientific Reports 2019;9. doi:10.1038/s41598-019-43110-z. 10.1038/s41598-019-43110-z PMC6484027 31024051

[57] Jurgens G., Saano A. (1999). Diversity of soil Archaea in boreal forest before, and after clear-cutting and prescribed burning. FEMS Microbiol Ecol.

[58] Tripathi BM, Kim M, Kim Y, Byun E, Yang JW, Ahn J, et al. Variations in bacterial and archaeal communities along depth profiles of Alaskan soil cores. Scientific Reports 2018;8. doi:10.1038/s41598-017-18777-x. 10.1038/s41598-017-18777-x PMC5765012 29323168

[59] Wei S, Cui H, He H, Hu F, Su X, Zhu Y. Diversity and distribution of archaea community along a stratigraphic permafrost profile from qinghai-tibetan plateau, China. Archaea 2014;2014. doi:10.1155/2014/240817. 10.1155/2014/240817 PMC4261641 25525409

[60] Walker CB, De La Torre JR, Klotz MG, Urakawa H, Pinel N, Arp DJ, et al. Nitrosopumilus maritimus genome reveals unique mechanisms for nitrification and autotrophy in globally distributed marine crenarchaea. Proceedings of the National Academy of Sciences of the United States of America 2010;107:8818–23. doi:10.1073/pnas.0913533107. 10.1073/pnas.0913533107 PMC2889351 20421470

[61] Bomberg Malin, Timonen Sari (2007). Distribution of cren- and euryarchaeota in scots pine mycorrhizospheres and boreal forest humus. Microb Ecol.

[62] Glaser Karin, Baumann Karen, Leinweber Peter, Mikhailyuk Tatiana, Karsten Ulf (2018). Algal richness in BSCs in forests under different management intensity with some implications for P cycling. Biogeosciences.

[63] Tedersoo Leho, Bahram Mohammad, Cajthaml Tomáš, Põlme Sergei, Hiiesalu Indrek, Anslan Sten (2016). Tree diversity and species identity effects on soil fungi, protists and animals are context dependent. ISME J.

[64] Degrune F, Dumack K, Fiore-Donno AM, Bonkowski M, Sosa-Hernández MA, Schloter M, et al. Distinct communities of Cercozoa at different soil depths in a temperate agricultural field. FEMS Microbiology Ecology 2019;95. doi:10.1093/femsec/fiz041. 10.1093/femsec/fiz041 30915436

[65] Fiore-Donno AM, Richter-Heitmann T, Degrune F, Dumack K, Regan KM, Marhan S, et al. Functional traits and spatio-temporal structure of a major group of soil protists (rhizaria: Cercozoa) in a temperate grassland. Frontiers in Microbiology 2019;10. doi:10.3389/fmicb.2019.01332. 10.3389/fmicb.2019.01332 PMC6579879 31244819

[66] Kamdem M.M., Voua Otomo P., Ngakou A., Njintang Yanou N. (2018). Distribution and diversity of earthworm (Annelida, Clitellata) populations across four land use types in northern Cameroon. Turk J Zool.

[67] Lo-Man-Hung Nancy F., Marichal Raphaël, Candiani David F., Carvalho Leonardo S., Indicatti Rafael P., Bonaldo Alexandre B. (2011). Impact of different land management on soil spiders (Arachnida: Araneae) in two Amazonian areas of Brazil and Colombia. Journal of Arachnology.

[68] Renco M., Cerevková A., Gömöryová E. (2019). Soil nematode fauna and microbial characteristics in an early-successional forest ecosystem. Forests.

[69] Southward Eve C., Schulze Anja, Gardiner Stephen L. (2005). Pogonophora (Annelida): Form and function. Hydrobiologia.

[70] Bleidorn Christoph, Helm Conrad, Weigert Anne, Aguado Maria Teresa (2015). Evolutionary Developmental Biology of Invertebrates 2.

[71] Griffiths R.I., Thomson B.C., James P., Bell T., Bailey M., Whiteley A.S. (2011). The bacterial biogeography of British soils. Environ Microbiol.

[72] Ren Baihui, Hu Yuanman, Chen Baodong, Zhang Ying, Thiele Jan, Shi Rongjiu (2018). Soil pH and plant diversity shape soil bacterial community structure in the active layer across the latitudinal gradients in continuous permafrost region of Northeastern China. Sci Rep.

[73] Xiong Jinbo, Liu Yongqin, Lin Xiangui, Zhang Huayong, Zeng Jun, Hou Juzhi (2012). Geographic distance and pH drive bacterial distribution in alkaline lake sediments across Tibetan Plateau. Environ Microbiol.

[74] Zhou Z, Wang C, Luo Y. Meta-analysis of the impacts of global change factors on soil microbial diversity and functionality. Nature Communications 2020;11. doi:10.1038/s41467-020-16881-7. 10.1038/s41467-020-16881-7 PMC7300008 32555185

[75] Zhao ZB, He JZ, Geisen S, Han LL, Wang JT, Shen JP, et al. Protist communities are more sensitive to nitrogen fertilization than other microorganisms in diverse agricultural soils. Microbiome 2019;7. doi:10.1186/s40168-019-0647-0. 10.1186/s40168-019-0647-0 PMC6393985 30813951

[76] Mahé F, De Vargas C, Bass D, Czech L, Stamatakis A, Lara E, et al. Parasites dominate hyperdiverse soil protist communities in Neotropical rainforests. Nature Ecology and Evolution 2017;1. doi:10.1038/s41559-017-0091. 10.1038/s41559-017-0091 28812652

[77] Bongiorno Giulia, Bodenhausen Natacha, Bünemann Else K., Brussaard Lijbert, Geisen Stefan, Mäder Paul (2019). Reduced tillage, but not organic matter input, increased nematode diversity and food web stability in European long-term field experiments. Mol Ecol.

[78] Huhe, Chen X, Hou F, Wu Y, Cheng Y. Bacterial and fungal community structures in Loess Plateau grasslands with different grazing intensities. Frontiers in Microbiology 2017;8. doi:10.3389/fmicb.2017.00606. 10.3389/fmicb.2017.00606 PMC5383705 28439265

[79] Rughöft Saskia, Herrmann Martina, Lazar Cassandre S., Cesarz Simone, Levick Shaun R., Trumbore Susan E. (2016). Community composition and abundance of bacterial, archaeal and nitrifying populations in savanna soils on contrasting bedrock material in Kruger National Park, South Africa. Front Microbiol.

[80] Sun X, Zhang X, Zhang S, Dai G, Han S, Liang W. Soil nematode responses to increases in nitrogen deposition and precipitation in a temperate forest. PLoS ONE 2013;8. doi:10.1371/journal.pone.0082468. 10.1371/journal.pone.0082468 PMC3855740 24324794

