# Supplementary material for: Differential response of soil microbial and animal communities along the chronosequence of Cunninghamia lanceolata at different soil depth levels in subtropical forest ecosystem
Source: J Adv Res. 2021 Aug 11;38:41–54. doi: 10.1016/j.jare.2021.08.005 (PMC9091736; doi:10.1016/j.jare.2021.08.005)

Captions for Supplementary Figures and Tables

Supplementary Figures Captions.

Supplementary Fig S1. Soil physical and chemical characteristics for all samples after pooling the replications. Here, D1, D2, D3 are about different soil depths i.e., D1 (0–10 cm), D2 (10–20 cm) and D3 (20–40 cm). Error bars in the boxplots with different lowercase letters show significant differences between different depths, while different uppercase letters describe significant differences among stand ages (LSD test). The values of two way ANOVA interactions have also been added (Red color text). Here, Significance codes are, 0 '***' 0.001 '**' 0.01 '*' 0.05 '.' 0.1 ' ' 1

Supplementary Fig S2. Relative abundance (%) of top 15 soil microbial (Bacteria, Fungi, Archaea, Protists) and animal communities for all samples. Here, 1,2,3 are for replication 1, replication 2 and replication 3, while, D1, D2, D3 are about different soil depths i.e., D1 (0–10 cm), D2 (10–20 cm) and D3 (20–40 cm).

Supplementary Fig S3. Selected relative abundance trend lines of top 15 soil microbial (Bacteria, Fungi, Archaea, Protists) and animal communities for all samples designated by (a, b, c, d, e). Different biotic community are designated with capital A,B,C,----etc. Here, different stand ages are listed at x-axis, while, D1 (Green color line), D2 (Blue color line), D3 (Brown color line) are about different soil depths i.e., D1 (0–10 cm), D2 (10–20 cm) and D3 (20–40 cm).

Supplementary Fig S4. Alpha diversity (Chao 1 and Shannon indices) of soil microbial (Bacteria, Fungi, Archaea, Protists) and animal communities. Here, different stand ages are listed at x-axis, while, D1 (Red color line), D2 (Green color line), D3 (Blue color line) are about different soil depths i.e., D1 (0–10 cm), D2 (10–20 cm) and D3 (20–40 cm). The values of two way ANOVA interactions have been added. Here, Significance codes are, 0 '***' 0.001 '**' 0.01 '*' 0.05 '.' 0.1 ' ' 1

Supplementary Fig S5. Correlation heatmap of soil microbial (Bacteria, Fungi, Archaea, Protists) and animal communities composition and environmental factors.

Table captions

Supplementary Table 1. Two way ANOVA interactions showing the effect of age, soil depth and combine effect of age & depth on relative abundance of top 15 microbial (Bacteria, Fungi, Archaea, Protists) and animal communities.

Supplementary Table 2. Two way permutational multivariate analysis of variance (PERMANOVA) (Adonis & ANOSIM analysis) showing the effects of age and soil depth on microbial (Bacteria, Fungi, Archaea, Protists) and animal community structure.

Supplementary Table 3. Summary of relationships amongst environmental factors and microbial (Bacteria, Fungi, Archaea, Protists) and animal communities.

Supplementary Fig S1.

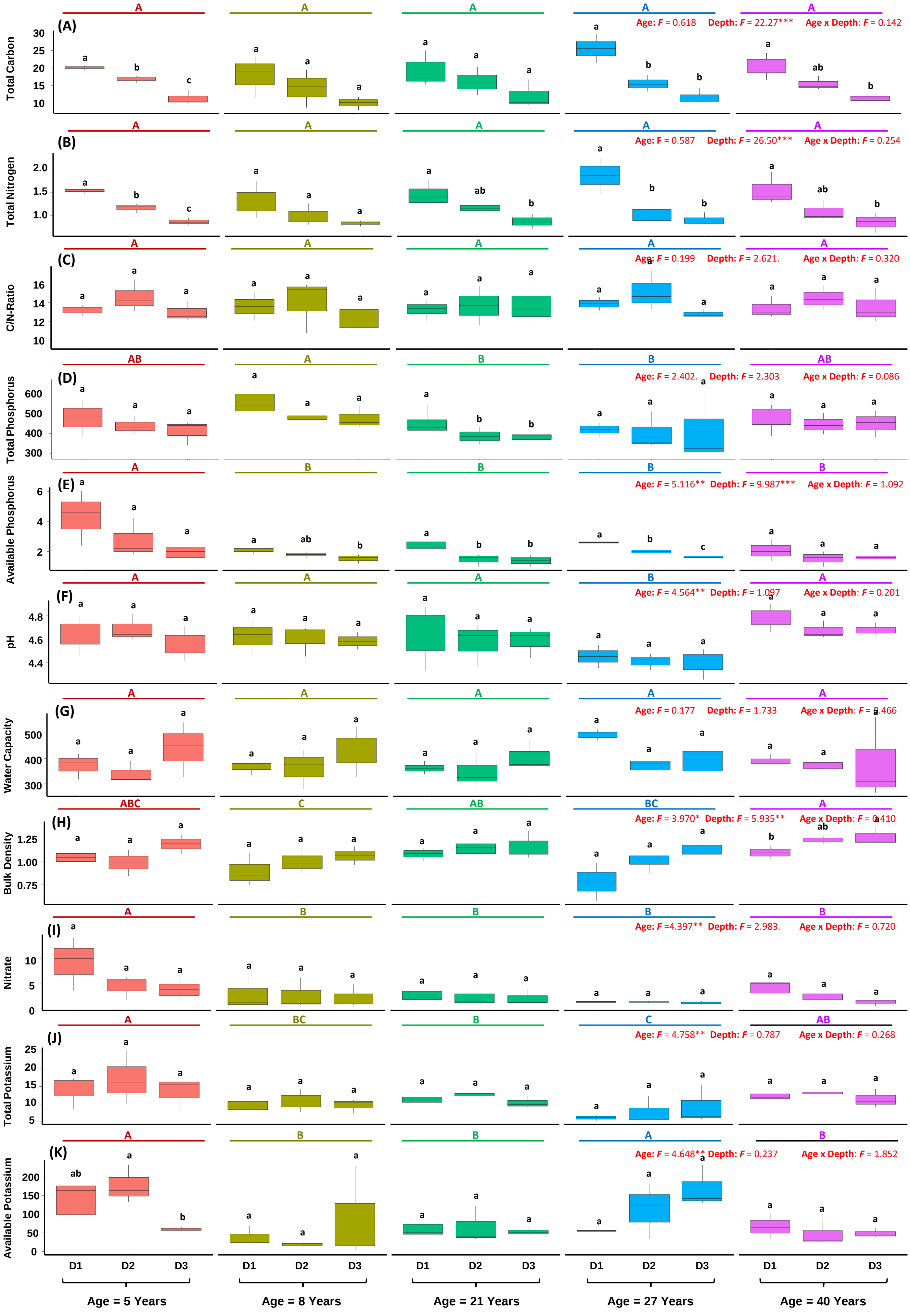

Supplementary Fig S2.

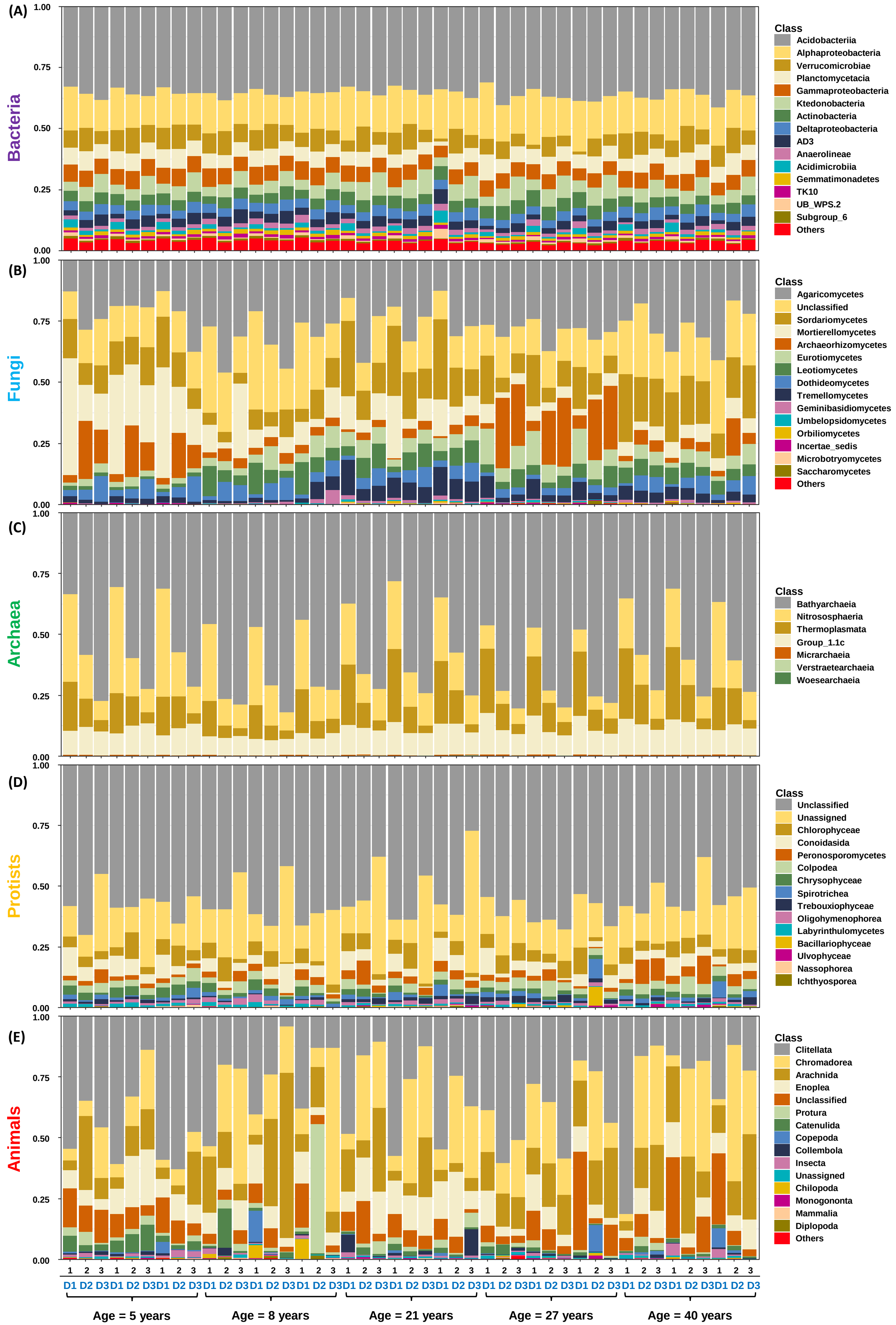

Supplementary Fig S3a.

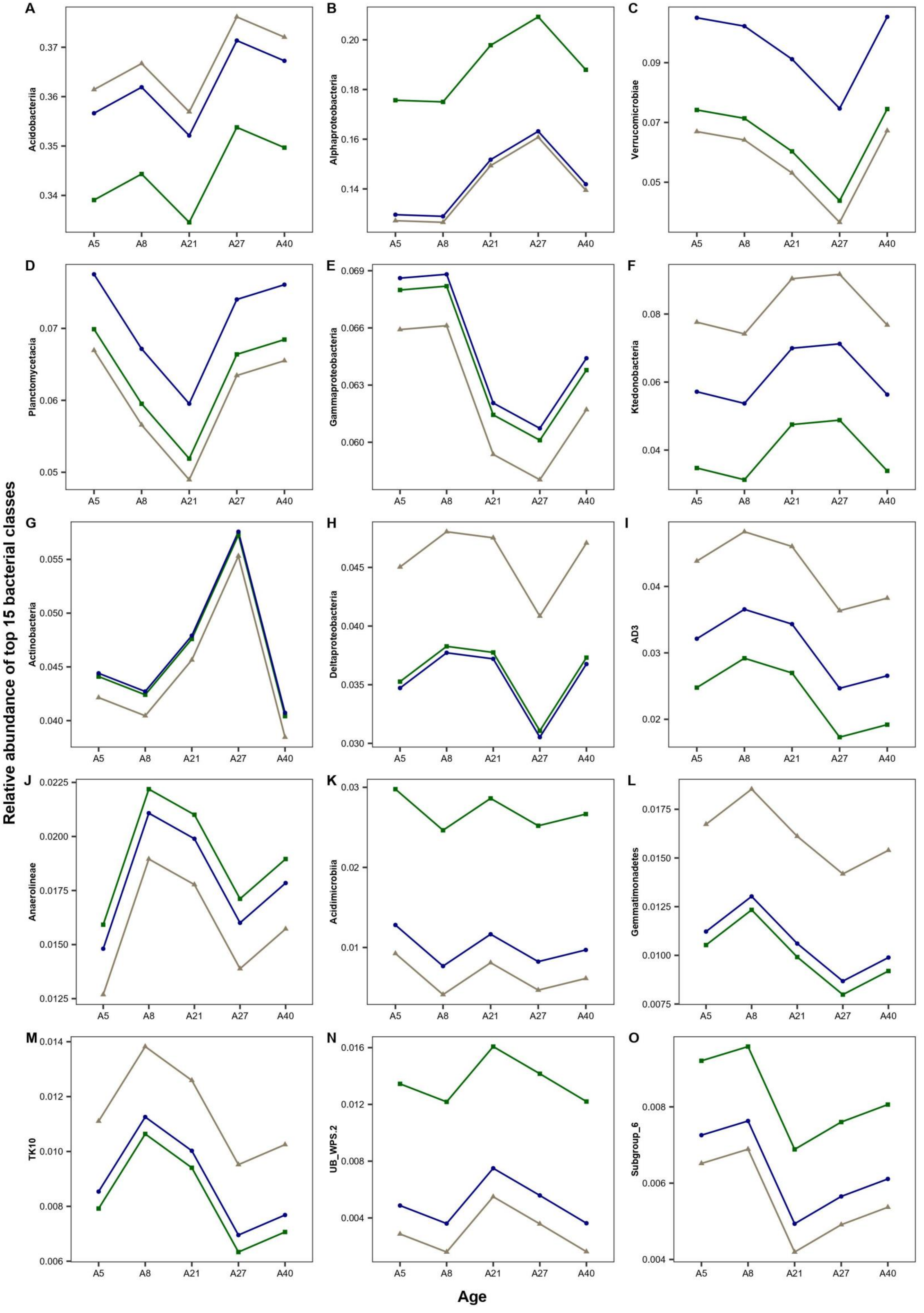

Supplementary Fig S3b.

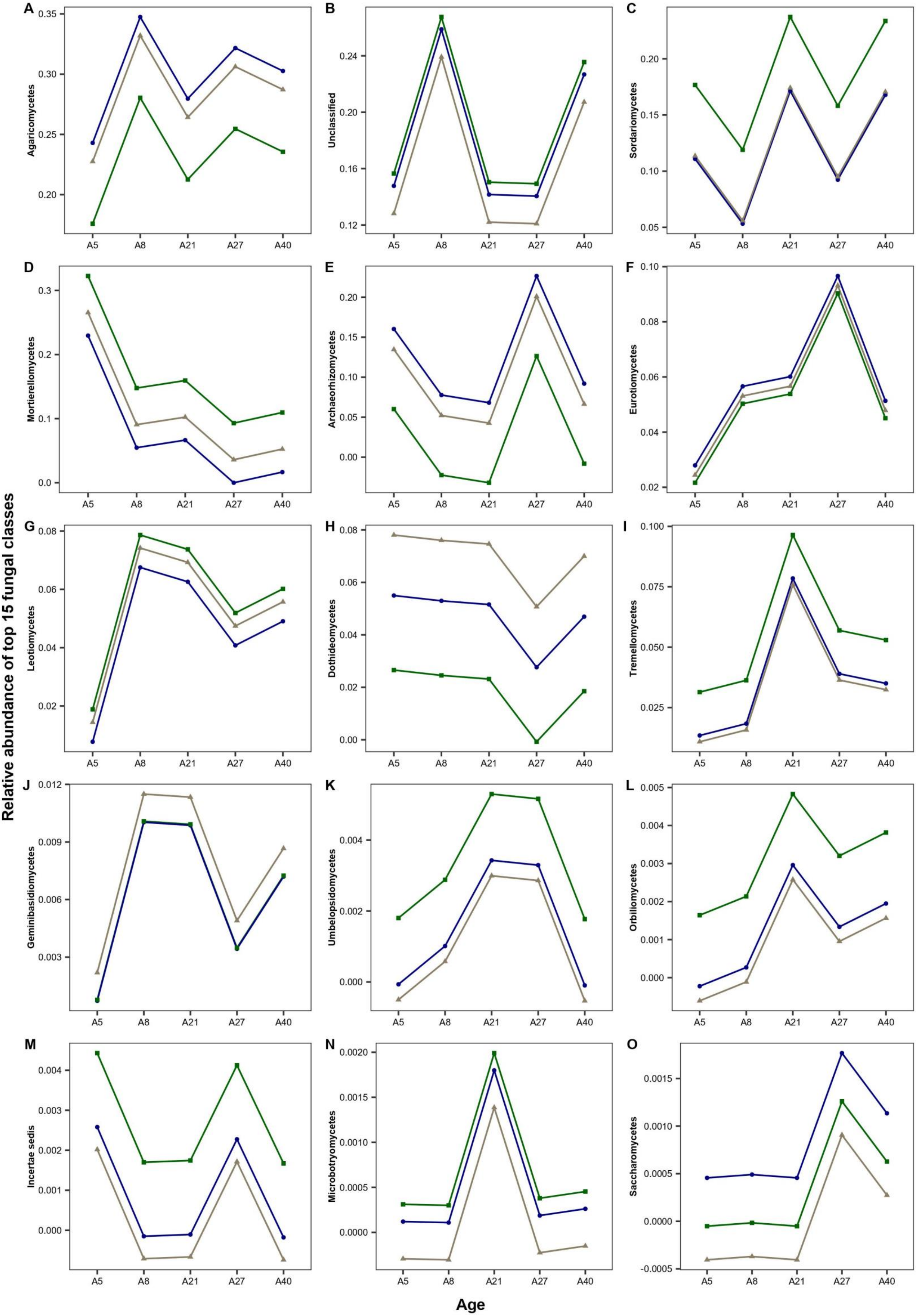

Supplementary Fig S3c.

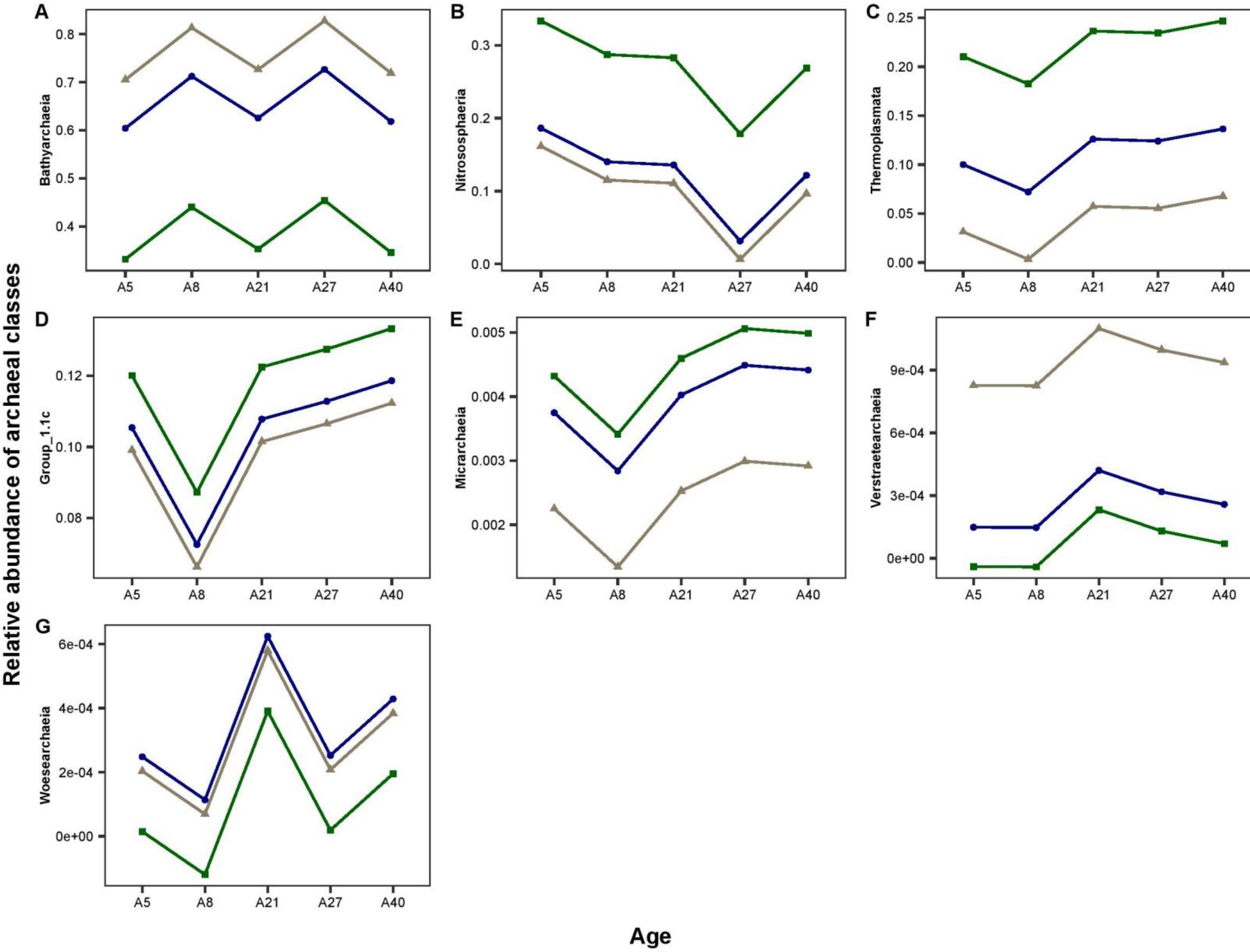

Supplementary Fig S3d.

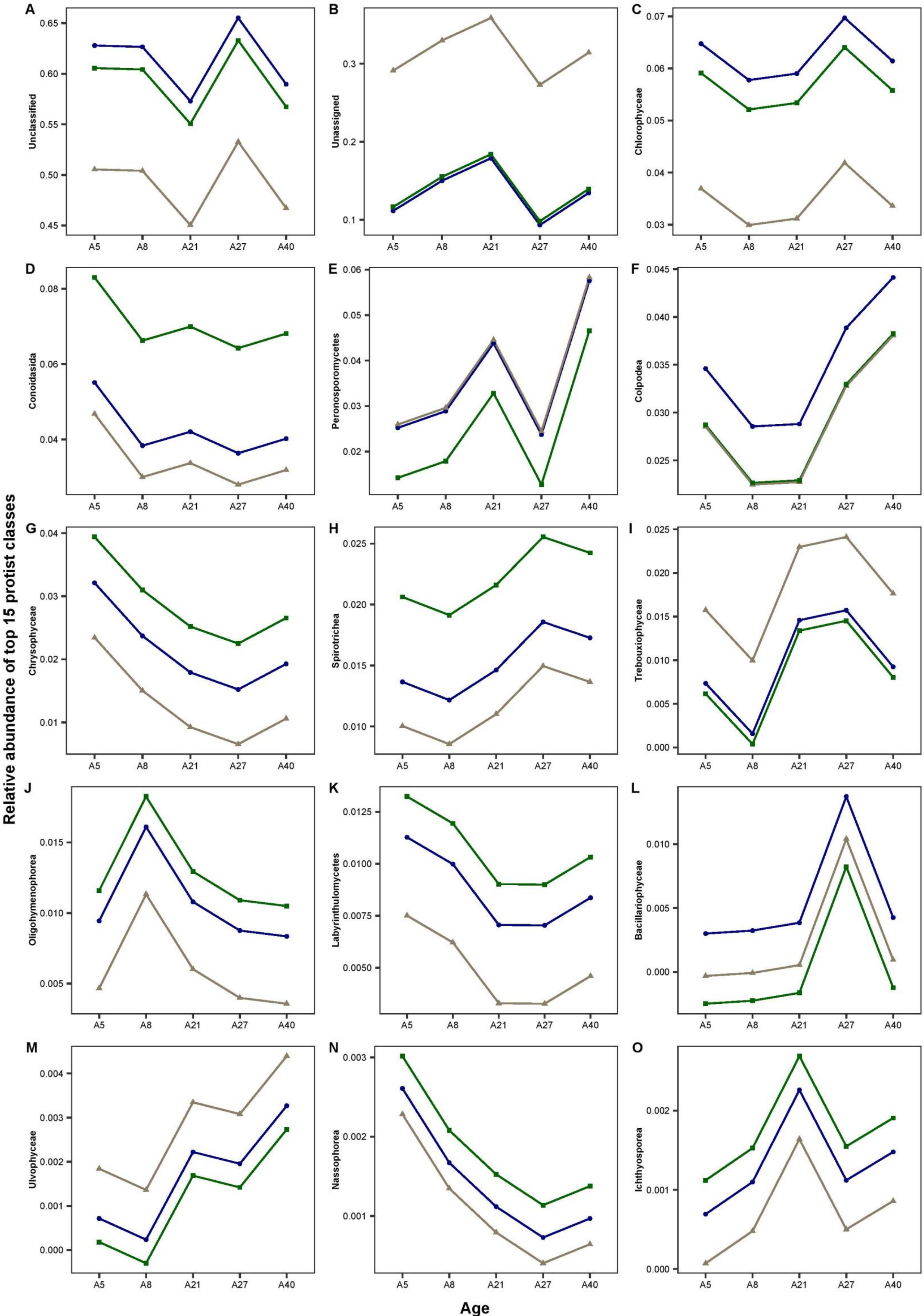

Supplementary Fig S3e.

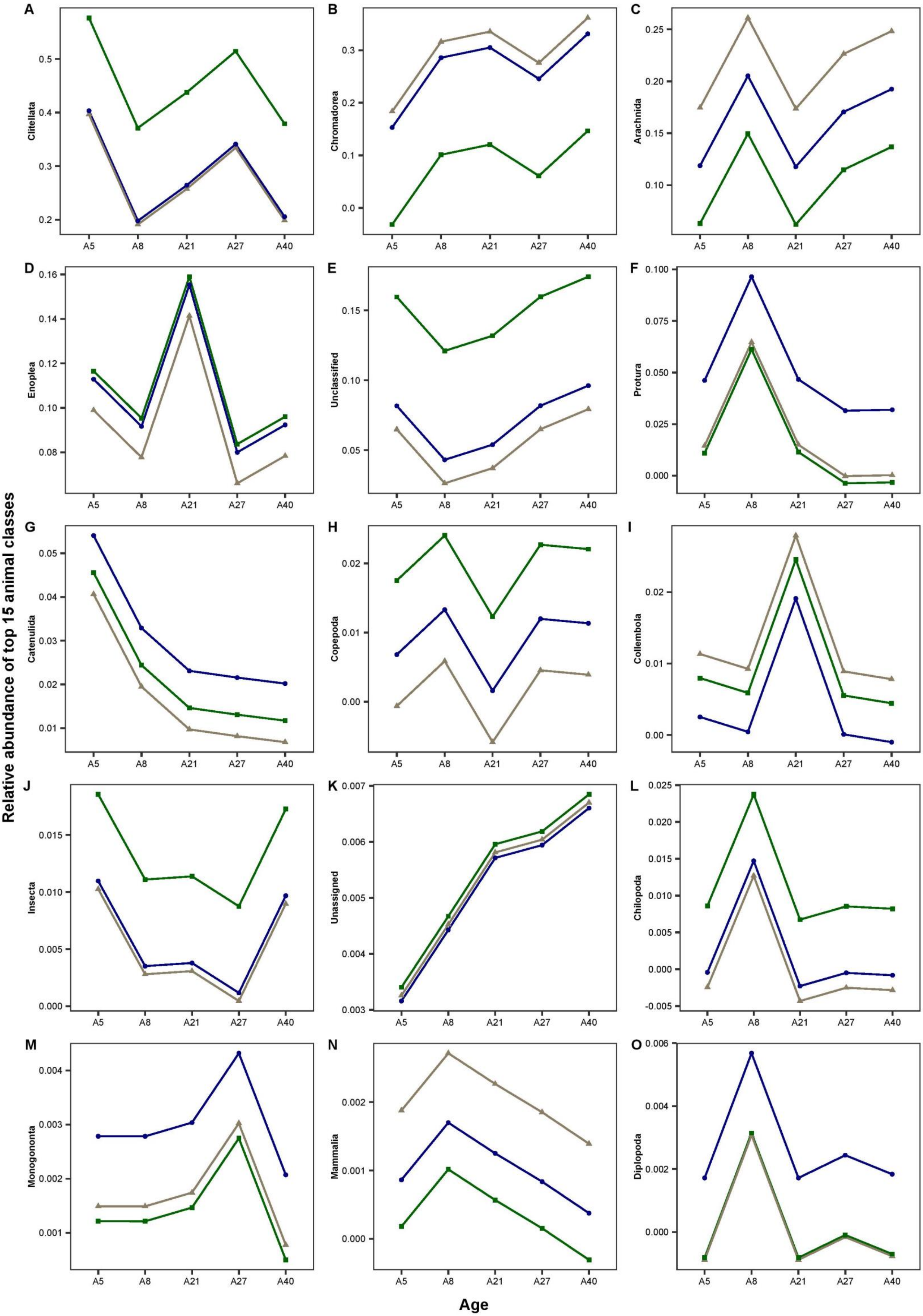

Supplementary Fig S4.

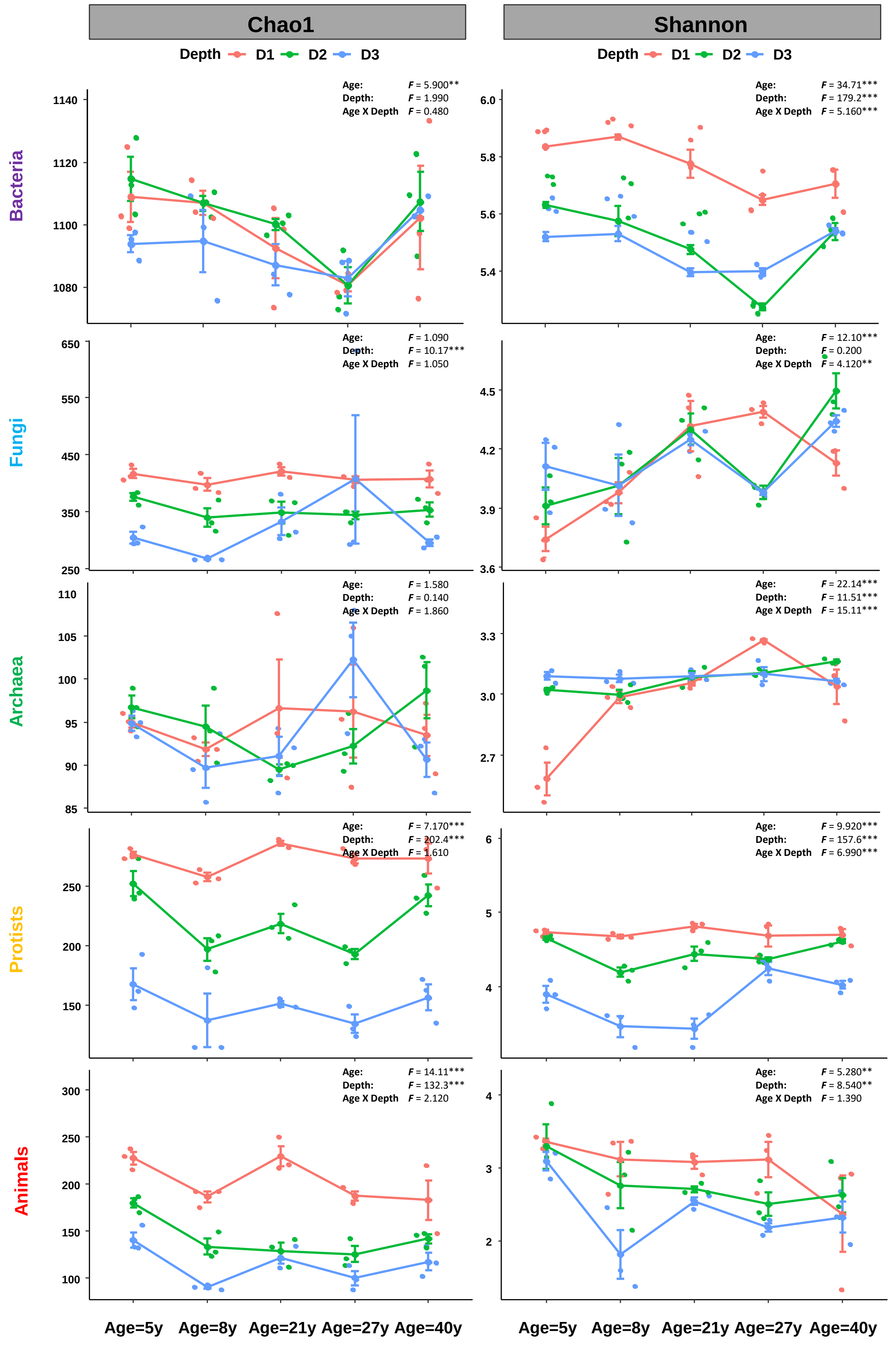

Supplementary Table 1

Biotic Communities	Class	Age			Depth			Age x Depth		
		F value	P value	Pr (>F)	F value	P value	Pr (>F)	F value	P value	Pr (>F)
Bacteria	Acidobacteriia	1.088	0.380		0.769	0.427		0.478	0.861	
	Alphaproteobacteria	2.208	0.091	.	0.003	0.996		0.312	0.955	
	Verrucomicrobiae	2.415	0.070	.	0.538	0.589		0.359	0.933	
	Planctomycetacia	3.455	0.019	*	0.230	0.795		0.730	0.664	
	Gammaproteobacteria	3.442	0.019	*	2.145	0.134		0.839	0.575	
	Ktedonobacteria	1.222	0.333		0.004	0.995		0.041	1.000	
	Actinobacteria	12.25	0.000	***	0.702	0.932		0.390	0.917	
	Deltaproteobacteria	1.988	0.121		0.138	0.871		0.279	0.967	
	AD3	1.522	0.220		0.789	0.828		0.185	0.991	
	Anaerolineae	2.515	0.062	.	1.395	0.263		0.424	0.897	
	Acidimicrobiia	0.297	0.877		0.021	0.979		0.273	0.969	
	Gemmatimonadetes	1.733	0.168		0.074	0.928		0.162	0.994	
	TK10	4.880	0.008	**	0.167	0.846		0.174	0.992	
	UB_WPS.2	0.457	0.766		0.308	0.736		0.373	0.926	
	Subgroup_6	2.711	0.048	*	0.347	0.709		0.170	0.993	
Fungi	Agaricomycetes	1.957	0.126		0.121	0.886		0.355	0.935	
	Unclassified	7.492	0.000	***	0.000	0.999		0.321	0.951	
	Sordariomycetes	5.758	0.001	**	0.094	0.910		0.303	0.958	
	Mortierellomycetes	9.903	0.000	***	0.077	0.925		0.270	0.970	
	Archaeorhizomycetes	7.509	0.000	***	0.035	0.964		0.092	0.999	
	Eurotiomycetes	7.197	0.000	***	0.255	0.776		0.470	0.866	
	Leotiomycetes	6.138	0.000	***	0.236	0.790		0.132	0.997	
	Dothideomycetes	1.291	0.296		0.005	0.994		0.136	0.996	
	Tremellomycetes	11.58	0.000	***	0.299	0.743		1.014	0.446	
	Geminibasidiomycetes	2.509	0.062	.	1.345	0.275		1.758	0.125	
	Umbelopsidomycetes	8.162	0.000	***	0.323	0.726		0.454	0.878	
	Orbiliomycetes	5.346	0.002	**	0.213	0.809		0.187	0.990	
	Incertae_sedis	5.737	0.001	**	0.020	0.979		0.023	0.999	
	Microbotryomycetes	9.048	0.000	***	0.565	0.573		0.404	0.908	
	Saccharomycetes	0.823	0.520		0.675	0.516		1.046	0.425	
Archaea	Bathyarchaeia	0.772	0.551		0.025	0.974		0.015	1.000	
	Nitrososphaeria	2.551	0.059	.	0.132	0.872		0.051	0.999	
	Thermoplasmata	0.651	0.630		0.010	0.989		0.081	0.999	
	Group_1.1c	4.904	0.003	**	0.043	0.957		0.288	0.964	
	Micrarchaeia	1.986	0.122		0.044	0.956		0.106	0.998	
	Verstraetearchaeia	0.378	0.822		0.085	0.918		0.423	0.897	
	Woesearchaeia	5.571	0.001	**	1.321	0.281		0.389	0.917	
Protists	Unclassified	3.202	0.026	*	20.99	0.000	***	3.428	0.006	**
	Unassigned	6.507	0.000	***	102.2	0.000	***	14.73	0.000	***
	Chlorophyceae	0.409	0.800		6.379	0.004	**	1.842	0.107	
	Conoidasida	1.010	0.417		11.04	0.000	***	2.196	0.056	.
	Peronosporomycetes	4.591	0.005	**	1.566	0.225		1.969	0.085	.
	Colpodea	2.824	0.042	*	1.262	0.297		1.937	0.090	.
	Chrysophyceae	10.66	0.000	***	25.96	0.000	***	2.664	0.024	*
	Spirotrichea	0.257	0.902		1.805	0.181		1.315	0.273	
	Trebouxiophyceae	14.00	0.000	***	19.35	0.000	***	2.514	0.031	*
	Oligohymenophorea	2.462	0.066	.	5.133	0.012	*	1.210	0.326	
	Labyrinthulomycetes	3.808	0.012	*	15.57	0.000	***	3.073	0.011	*
	Bacillariophyceae	1.334	0.280		0.817	0.451		0.811	0.597	
	Ulvophyceae	2.184	0.094	.	1.772	0.187		1.354	0.255	
	Nassophorea	1.272	0.302		0.508	0.606		1.126	0.374	
	Ichthyosporea	1.225	0.320		1.631	0.212		1.188	0.338	
Animals	Clitellata	3.770	0.013	*	8.340	0.001	**	2.385	0.040	*
	Chromadorea	4.918	0.003	***	23.01	0.000	***	4.100	0.002	**
	Arachnida	0.945	0.451		2.926	0.069	.	0.307	0.957	
	Enoplea	3.450	0.019	*	0.560	0.577		1.684	0.143	
	Unclassified	0.809	0.528		7.139	0.002	**	0.913	0.518	
	Protura	1.077	0.385		0.948	0.398		1.142	0.364	
	Catenulida	2.149	0.099	.	0.821	0.449		0.645	0.733	
	Copepoda	0.260	0.900		1.565	0.225		0.881	0.543	
	Collembola	1.758	0.163		0.819	0.450		0.819	0.591	
	Insecta	2.830	0.041	*	5.501	0.009	**	3.569	0.005	**
	Unassigned	1.619	0.195		0.021	0.978		0.209	0.055	.
	Chilopoda	6.675	0.000	***	7.705	0.001	**	7.773	0.000	***
	Monogononta	1.413	0.253		2.457	0.102		1.231	0.315	
	Mammalia	1.731	0.169		8.543	0.001	**	0.711	0.679	
	Diplopoda	23.78	0.000	***	30.03	0.000	***	20.80	0.000	***

Significance codes here :- 0 '*' 0.001 '**' 0.01 '*' 0.05 '.' 0.1 ' ' 1**

Supplementary Table 2

Biotic Communities	Variable	ANOSIM		ADONIS		Stress
		R	*p*-value	R^2	*p*-value	
**Bacteria**	Age	0.1671	0.001	0.2089	0.001	0.034279
	Depth	0.7940	0.001	0.5381	0.001	
**Fungi**	Age	0.7094	0.001	0.4411	0.001	0.069439
	Depth	0.3671	0.001	0.1876	0.001	
**Archaea**	Age	0.2127	0.001	0.2455	0.001	0.036044
	Depth	0.7869	0.001	0.5718	0.001	
**Protists**	Age	0.2762	0.001	0.2297	0.001	0.089382
	Depth	0.5050	0.001	0.2588	0.001	
**Animals**	Age	0.1803	0.001	0.1662	0.001	0.099358
	Depth	0.3108	0.001	0.1494	0.001	

Supplementary Table 3

Physiochemical properties	Bacteria			Fungi			Archaea			Protists			Animals		
	F-value	p-value	Significance Level	F-value	p-value	Significance Level	F-value	p-value	Significance Level	F-value	p-value	Significance Level	F-value	p-value	Significance Level
TN	11.734	0.001	***	5.051	0.002	**	53.004	0.001	***	7.579	0.001	***	7.754	0.001	***
TP	2.875	0.027	*	2.273	0.050	*	4.191	0.031	*	0.725	0.559		0.898	0.476	
AP	0.188	0.967		6.552	0.001	***	4.315	0.028	*	0.685	0.587		2.699	0.027	*
pH	4.010	0.010	**	4.621	0.002	**	15.575	0.001	***	0.948	0.418		1.590	0.140	
WC	1.467	0.196		0.774	0.538		0.408	0.618		2.436	0.075	.	0.670	0.664	
BD	0.773	0.544		3.035	0.018	*	0.808	0.418		1.757	0.116		1.120	0.300	
NO_3	2.083	0.093	.	3.568	0.004	**	2.863	0.087	.	0.896	0.431		1.33	0.242	
TK	1.530	0.192		1.590	0.181		0.819	0.425		0.504	0.77		0.276	0.970	
AK	0.980	0.395		0.012	0.012	*	0.067	0.954		2.200	0.074	.	2.163	0.062	.
**Model**	**2.849**	**0.001**	***	**3.415**	**0.001**	***	**9.117**	**0.001**	***	**1.970**	**0.011**	*	**2.056**	**0.001**	***
**RDA1**	**13.981**	**0.001**	***	**13.691**	**0.001**	***	**80.186**	**0.001**	***	**12.498**	**0.014**	*	**12.454**	**0.001**	***
**RDA2**	**9.597**	**0.038**	*	**9.797**	**0.001**	***	**5.637**	**0.436**		**2.105**	**0.934**		**2.440**	**0.0845**	

Supplementary Fig S5.

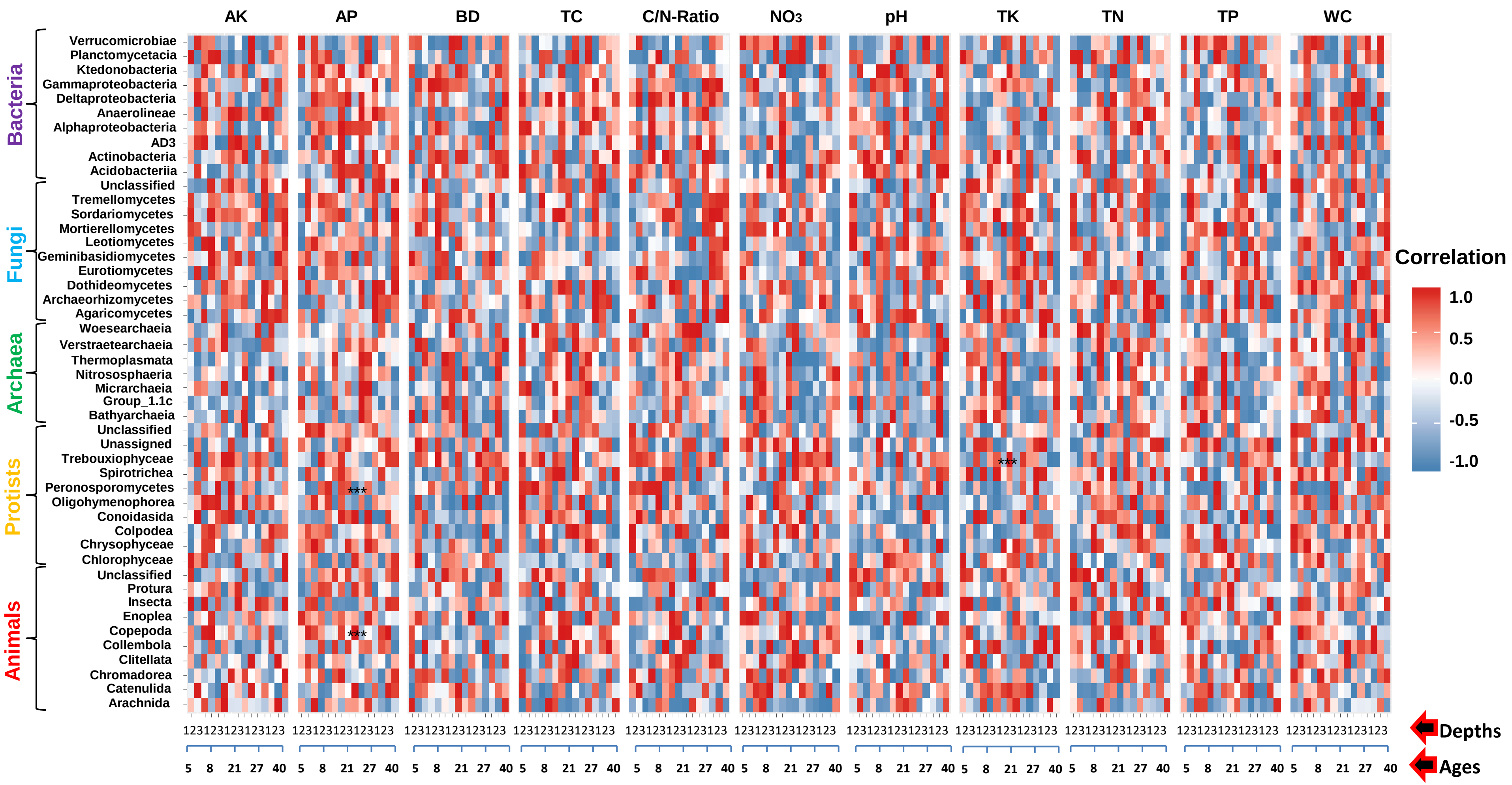

Supplement: Supplementary data 1 [file mmc1.pdf]
